# Amplification of Secondary Flow at the Initiation Site of Intracranial Sidewall Aneurysms

**DOI:** 10.1007/s13239-025-00771-4

**Published:** 2025-01-27

**Authors:** Benjamin Csippa, Péter Friedrich, István Szikora, György Paál

**Affiliations:** 1 Department of Hydrodynamic Systems, Faculty of Mechanical Engineering, Budapest University of Technology and Economics, Műegyetem rkp. 1-3, Budapest, 1111 Hungary; 2 Department Neurointerventions, Semmelweis University, Department for Neurosurgery and Neurointerventions, Amerikai út 57., Budapest, Hungary

**Keywords:** Intracranial aneurysm, Initiation, Secondary flows, CFD, VMTK

## Abstract

**Purpose:**

The initiation of intracranial aneurysms has long been studied, mainly by the evaluation of the wall shear stress field. However, the debate about the emergence of hemodynamic stimuli still persists. This paper builds on our previous hypothesis that secondary flows play an important role in the formation cascade by examining the relationship between flow physics and vessel geometry.

**Methods:**

A composite evaluation framework was developed to analyze the simulated flow field in perpendicular cross-sections along the arterial centerline. The velocity field was decomposed into secondary flow components around the centerline in these cross-sections, allowing the direct comparison of the flow features with the geometrical parameters of the centerline. Qualitative and statistical analysis was performed to identify links between morphology, flow, and the formation site of the aneurysms.

**Results:**

The normalized mean curvature and curvature peak were significantly higher in the aneurysmal bends than in other arterial bends. Similarly, a significant difference was found for the normalized mean velocity ($p=0.0274$), the circumferential ($p=0.0029$), and radial ($p=0.0057$) velocity components between the arterial bends harboring the aneurysm than in other arterial bends. In contrast, the difference of means for the normalized axial velocity is insignificant ($p=0.1471$).

**Conclusion:**

Thirty cases with aneurysms located on the ICA were analyzed in the virtually reconstructed pre-aneurysmal state by an *in-silico* study. We found that sidewall aneurysm formation on the ICA is more probable in these arterial bends with the highest case-specific curvature, which are accompanied by the highest case-specific secondary flows (circumferential and radial velocity components) than in other bends.

**Supplementary Information:**

The online version contains supplementary material available at 10.1007/s13239-025-00771-4.

## Introduction

The formation of intracranial aneurysms is associated with pathological changes inside the vessel wall. Even though the process has not been completely described until now, hemodynamics, atherosclerosis, hypertension, family history, and smoking are commonly associated with aneurysm formation [1]. As most of these epidemiological risk factors have a longer span of changing systemic alterations, aneurysms are rarely prevalent in the younger population. Hemodynamic stresses, like elevated wall shear stress or disturbed oscillatory flow, can act as mechanical stimuli to induce this process. Today, this research topic still attracts huge interest, connecting the numerical and medical communities even closer with ever-growing technological advancements.

Therefore, interest in understanding the aforementioned hemodynamic stimuli developed in the numerical community when appropriate techniques emerged. Table 1 showcases the evolution of the research in this field, but more information can be found in the review articles published over the last decade [1, 2]. The late start of the research in this field is partly because vessel imaging on a pre-aneurysm state is rare, mostly incidental, even today, with the increased amount of screening in hospitals. Thus, mostly aneurysmal geometries are used, and researchers developed methods to remove the aneurysm sac to reconstruct the healthy segment virtually. One of the earliest studies was performed by Mantha et al. [3]; they formulated the aneurysm formation index (AFI), a WSS metric for quantifying the change in WSS angle between the mid-systolic deceleration WSS vector and the cycle-averaged one. They argued that stagnation zones could be detected by the AFI and found strong secondary flows at the formation site. Others arrived at similar conclusions, using WSS divergence (WSSD) [4], isolated fluid impingement regions [5], and the emergence of complex flow structures due to decaying turbulence during systolic deceleration [6]. They emphasized that disturbed secondary flows may be associated with the initiation site. Animal experiments were done on dogs and rabbits with corresponding hemodynamic analysis, investigating the locations of interest [7, 8]. They found that elevated WSS and positive WSS gradients (WSSG) are present at the apex of these arterial bifurcations. Their findings were validated and even extended by other authors. In 2011 [9], our research group found two de-novo aneurysms located at different aneurysm-prone sites, and the hemodynamic analysis concluded that the same focal WSS and WSSG-mediated insult is also present in human vasculatures. Lauric et al. [10–12] also found a correlation with these metrics. Still, they were the first ones to emphasize the curvature effect and found that low and oscillatory WSS distribution resides next to the high WSS and WSSG regions. Shimogonya et al. [13, 14] developed another WSS metric, the gradient oscillatory number (GON). It was intended to quantify the degree of oscillating tension/compression forces acting on the wall. The analysis included only one geometry, and others found contradicting results on this metric [15, 16].

**Table 1:** A brief summary of the literature about aneurysm initiation

Author	IA type	Metrics	Cases (controls)
Mantha [3]	ICA	OSI, AFI	3
Meng [7]	artificial	WSS, WSSG	6**
Shimogonya [13]	ICA	WSS, WSSG, OSI, AFI,GON	1
Metaxa [8]	BA	WSS, WSSG	11**
Kulcsár [9]	BA, ACA, ICA	WSS, WSSG	3*
Lauric [10, 11]	ICA	WSS, WSSG	2
Sendstad [6]	ICA	Velocity fluctuations	5(5)
Chen [15]	ICA, MCA	WSS, OSI, AFI, GON	22
Geers [16]	ACA (A1)	TAWSS, TAWSSG, transWSS, WSSPI	10(10)
Ricardello [5]	ICA	Impingment	8(8)
Watanabe [17]	ICA	WSS, WSSG, nh-WSS, nh-WSSG	15(15)
Sunderland [18]	ICA	WSS, WSSG, AFI, GON, MV	18
Rosato [12]	ICA	WSS, WSSG, pressure	10(10)
Zimny [19]	MCA	WSS, WSSG, OSI	38(39)
Fujimura [4]	ICA, ACA, MCA, BA, VA	WSSD	10*

The metrics are defined in the text. *Means a de novo aneurysm, **means an animal study. All the other studies used arterial sections with a virtually removed aneurysm

Geers et al. [16] investigated 10 cases of aneurysms located on the A1 segment of the anterior cerebral artery (ACA) with 10 healthy control cases. This study was systematically designed to avoid discrepancies in patient selection and investigated the WSS metrics developed previously. They further validated the so-called high-flow theory (high WSS and WSSG) for bifurcation aneurysms and proposed other metrics that do not rely on derivatives of the WSS vector, the transWSS, and the WSS pulsatility index (WSSPI). Watanabe et al. [17] incorporated 15 subjects with aneurysms on one side, used the contralateral healthy ICAs, and further control cases from patients that had an aneurysm elsewhere. They evaluated the normalized highest WSS and WSSG (nh-WSS, nh-WSSG) points, and a receiver operating characteristic (ROC) curve analysis resulted in significantly different threshold values for nh-WSS and nh-WSSG (6.34 and 22.96, respectively) between aneurysmal and control groups. More advanced statistical methods were proposed by Sunderland and Zimny [18, 19], indicating the need for multivariate regression analysis. They obtained higher area under the curve (AUC) values from the ROC analysis using multivariate logistic regression methods incorporating multiple hemodynamic metrics. Table 1 displays that the focus was on the evaluation of WSS-related metrics, without a surprise, since mechanical stimuli acting on the endothelial layer arise from the wall shear stress. However, the numerical computation of WSS imposes some challenges. Medical image segmentation and subsequent smoothing of the resulting vessel surface are operator-dependent and highly subjective, leading to improper geometry representations [20, 21], which might partly explain the confounding result existing in the literature. On the other hand, Meng et al. [22] present in a review that these contradictory conclusions on high-vs-low WSS theories are not necessarily the result of improper modeling assumptions but rather that of the intrinsic mechanistic complexity concerning intracranial aneurysms.

Although the WSS field transports the mechanical stimuli acting on the vessel wall, the calculation of WSS over the complex triangulated shapes generated from segmentation can be highly uncertain. In-depth flow field analysis is rare, with the goal of explaining aneurysm initiation. Yet, it is often stated that disturbed and irregular flow patterns emerge at the usual locations of sidewall aneurysm formation [3–6, 11]. In the 2012 editorial, Professor Kallmes [23] pressed that only meaningful information should be extracted from CFD simulations. Our aim is not to formalize new metrics but to understand the underlying flow physics that will induce the mechano-signaling WSS field along the vessel wall.

In our pilot study [24], we formulated the hypothesis that secondary flows emerge and surge in the mid-systolic deceleration range at the later formation sites of sidewall aneurysms. Now, we want to step back a little to understand the complex flow physics first, and therefore, we focus on the time-averaged results to understand the mechanistic behavior of secondary flows.

In the present study, we analyze virtually reconstructed pre-aneurysmal flow fields of 30 patients using a composite evaluation technique to understand the secondary flows in order to shed some light on the connection between vessel geometry and flow.

## Methods

### Case Selection

Medical images of thirty patients acquired by DSA (Digital subtraction angiography) imaging (GE-Innova IGS 630) were provided by our medical partner. Data collection was approved by the hospital’s ethical committee, and written consent was obtained from all the patients. Cases from only the ICA region were selected with an aneurysm located after the clinoid bend and before the bifurcation into the middle and anterior cerebral artery.

### Geometric Modeling and Characterization

**Fig. 1:**
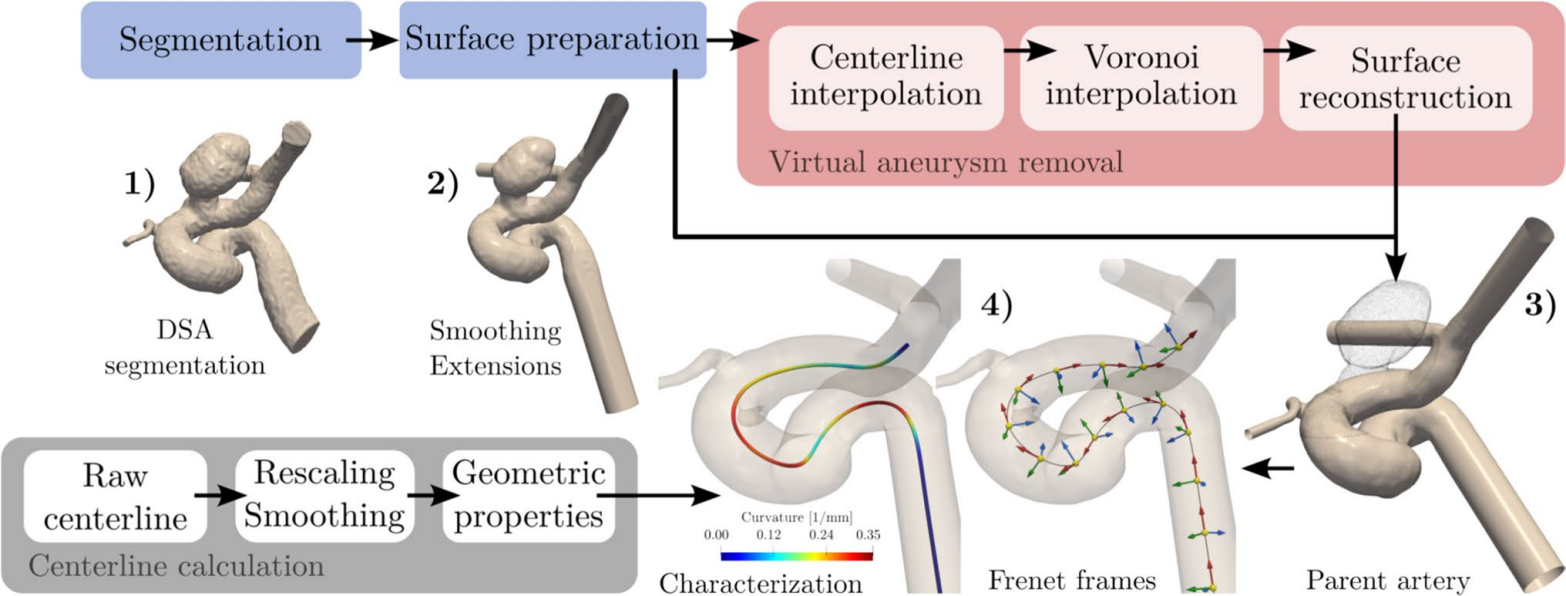
Geometrical modeling and centerline calculation. Top: Numerical geometry is created by the virtual removal of the aneurysm dome. Bottom: Centerline calculation and characterization as a 3D curve

DSA images were segmented in Slicer [25] with threshold initialization and subsequent manual editing. The raw triangulation after segmentation (See 1) in Fig. 1) was smoothed with the Taubin algorithm. Vessel boundaries were extended adaptively by an eight-diameter length for proper boundary condition development. Finally, the surface triangulation was remeshed to generate appropriate triangle sizes for later numerical meshing (See 2) in Fig. 1).

The literature summary table (Table 1) clearly shows that most of the studies resorted to a virtual removal technique to reconstruct the healthy parent vessel. Virtual removal is needed since imaging of the healthy vessel before aneurysm formation is rare and incidental, usually captured only when the patient has another related disease that needs angiography. Various methods exist for digital aneurysm removal. In this study, we opted to use an objective algorithm developed by Ford et al. [26], already implemented in the Vascular Modelling Toolkit (VMTK) [27]. The algorithm relies on the calculation of centerlines to generate an interpolation to reconstruct the healthy parent-vessel shape. After shape reconstruction, the surface geometry can be prepared for numerical meshing using the same triangle remeshing procedure mentioned above.

Tubular structures, like arterial vessels, can be described as $2\frac{1}{2}$D structures using a centerline curve and other intrinsic properties defined using the abscissa as a parameter of the space curve. In this work, the VMTK package was used to calculate centerlines [28]. This geometric entity and the associated properties, defined by the 3D curve representing the centerlines, are used in several tasks of this study. Therefore, centerlines and associated properties are described here so that terms and definitions later will be comprehensible. The centerline calculation of tubular structures can be defined as the computation of a curve from one extremity to another while maximizing the distance from the surface boundary along the path. For triangulated surfaces, the implemented method in the VMTK package relies on the calculation of the embedded Voronoi diagram. By definition, [27] the centerline lies on the Voronoi diagram and travels through the vertices with the largest maximum inscribed sphere radius. Numerically, the problem is equivalent to a minimum cost-path problem along the Voronoi diagram between a source and a target point.

The centerline in this form is a piecewise linear function $\textbf{C}(s)$ (s being the abscissa parameter along the arclength) connecting the vertices of the polygons along the Voronoi diagram. Therefore, because of the discretized definition of the Voronoi diagram, the centerline in its present state is highly affected by noise. A moving-average filter was used to smooth the centerline, implemented in VMTK to counter the noise. Smoothing is defined via the smoothing factor, and the number of iterations is fed through command-line arguments to the script. Furthermore, centerline rescaling was performed, as equal spacing will be needed later in the evaluation method. In this work, the division length was selected to be 0.15 mm, a smoothing factor of 1.0 with 75 iterations, following the recommendations of [29]. Smoothing and rescaling were needed because the associated properties of the centerline used later in the evaluation need the first, second, and third derivatives of the centerlines $\textbf{C}(s)$. The Frenet-Serret formulas define the derivatives of the unit vectors of the moving reference frames in terms of each other.

1 $$\begin{aligned} \frac{d \textbf{t}_f}{ds} = \kappa \textbf{n}_f, \frac{d \textbf{n}_f}{ds} = -\kappa \textbf{t}_f + \tau \textbf{b}_f, \frac{d \textbf{b}_f}{ds} = -\tau \textbf{n}_f \end{aligned}$$

where $\frac{d}{ds}$ is the derivative with respect to the arc length, $\kappa$ is the curvature, and $\tau$ is the torsion of the curve. $\textbf{t}_f$, $\textbf{n}_f$, and $\textbf{b}_f$ are the tangent, normal, and binormal unit vectors of the Frenet-frame, respectively, that form an orthonormal coordinate reference frame (See 4) in Fig. 1. The curvature measures the deviation of the 3D curve from a straight line, while the torsion measures the non-planarity of the curve or how suddenly the line twists in space. These properties strongly affect the locally emerging flow field. One of the objectives of this work is to find a link between the geometry and flow through these morphological properties of the blood vessels.

### Blood Flow Modelling

CFD calculations were carried out by ANSYS CFX (Ansys Inc., Canonsburg, PA, USA). The Navier–Stokes and continuity equations were solved with a cell-centered finite volume method. The second-order backward Euler scheme was selected for time integration with adaptive time-stepping to ensure a proper Courant number criterion ($CFL\le 0.8$). All the lumen geometries were discretized between 2.5 to 3 million numerical elements: on average, they consist of approximately 3 million, mostly tetrahedral cells, including six prismatic boundary layer cells adjacent to the vessel wall. Vessel walls were assumed to be rigid since we had no information on the local material properties of the surrounding supporting tissues, and in consideration of the large artery sizes, blood was approximated as an incompressible Newtonian fluid with the dynamic viscosity of $3.4\times 10^{-3} ~Pa\,s$ and density of $1055\, kg/m^3$.

**Fig. 2:**
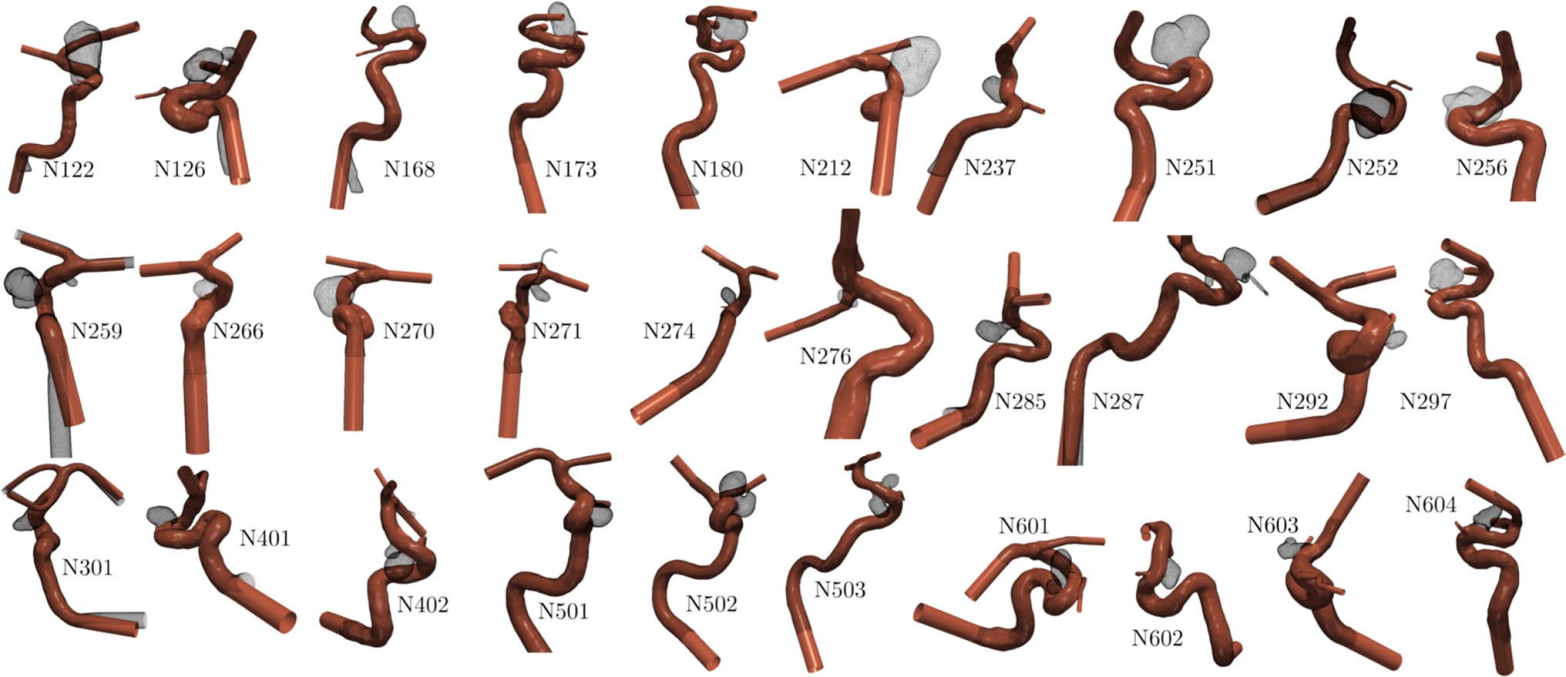
Cardiovascular models constructed for the study. The virtually reconstructed vessel can be seen with the wireframe of the removed aneurysm overlayed. Due to visualization purposes, the scaling is not uniform.

A time-varying parabolic velocity profile was imposed for the inlet boundary condition according to the waveform from [30]. The waveform was patient-specifically scaled, based on the cycle-averaged flow rate equation $Q = 48.21\,A^{1.84}\,T^{-1}$ obtained by [31]. Based on the period of the cardiac *T*[*s*] cycle and the inlet cross-section $A[cm^2]$, the flow rate *Q*[*ml*/*s*] can be calculated. Flow rate splitting boundary conditions were set on the outlets according to a modified Murray’s law [32] formulation. On the smallest outlet, 0 Pa static pressure was used. Three pulsatile cycles (T = 0.8 s) were simulated, totaling 2.4 s. Two hundred time steps were exported from the solution of only the last heartbeat cycle to remove any initial transients. Vascular geometries of the thirty cases can be seen in Fig. 2.

### Hemodynamics Along the Vessel

#### Frenet-frames

The results of the simulations for all thirty cases were exported for post-processing. A Python-based framework was developed to use the centerline information for the evaluation, using automated ParaView scripts. The proposed method relies on defining the centerlines and their properties similarly to [33], most notably the definition of the three unit vectors along the centerline points (see section “Geometric modeling and characterization” for description).

**Fig. 3:**
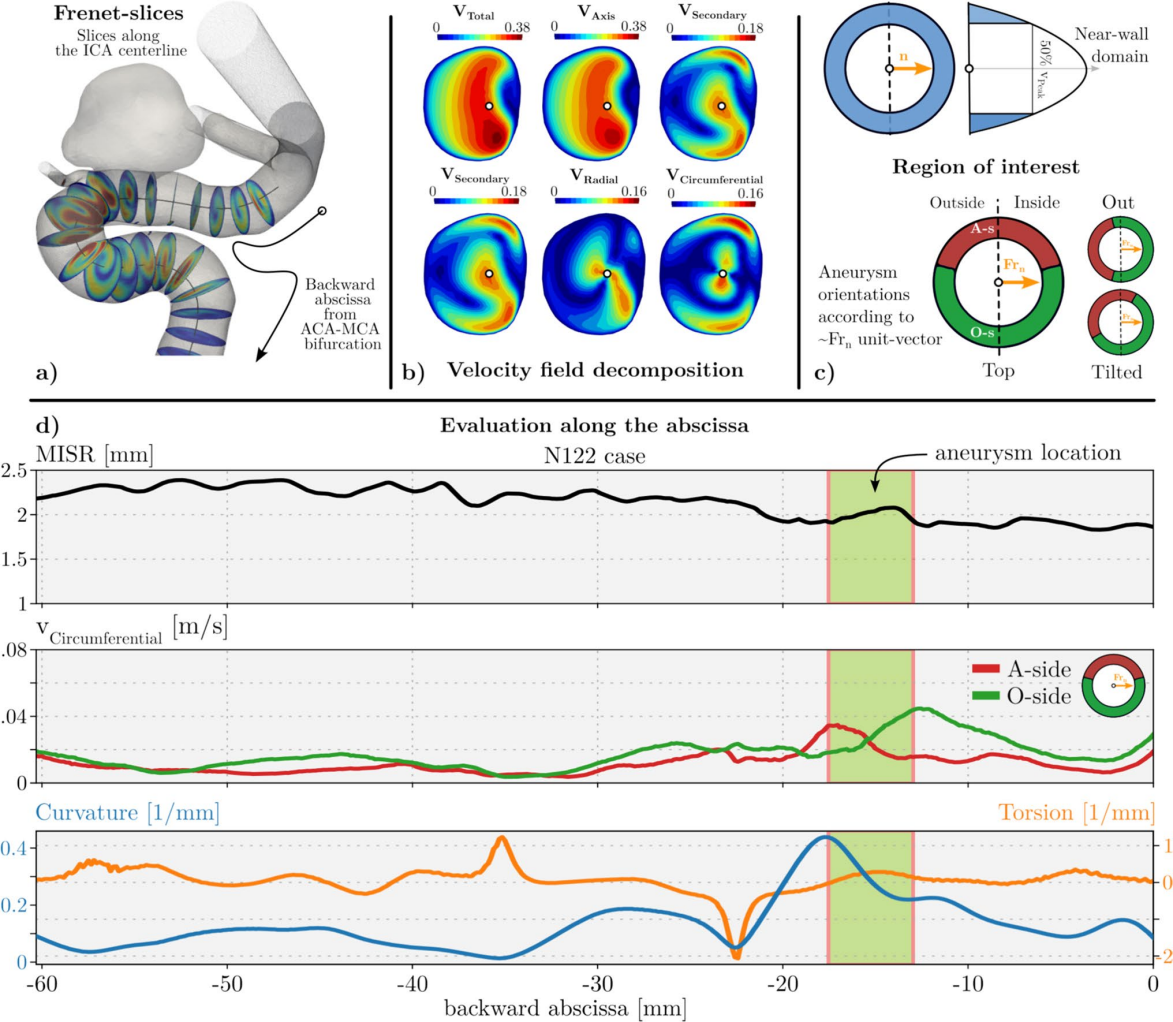
Evaluation framework for flow-field analysis along the centerline. **a** Perpendicular slices along the centerline are defined using the tangent unit vector of local Frenet-frames. **b** Velocity field decomposition into a local polar coordinate system. **c** Region of interest for the evaluation of near-wall flows. **d** Example diagram for comparing geometrical and flow field variables along the centerline. Top: maximum inscribed sphere radius (MISR). Middle: Circumferential velocity component on the aneurysmal and opposing side. Bottom: curvature $\kappa$ and torsion $\tau$ along the centerline. The aneurysm location is indicated by the green-shaded region. The horizontal axis represents the abscissa along the vessel section starting from the terminal bifurcation of the ICA. Negative values were used to showcase the direction of the blood flow.

The first step of the method is to define plane slices perpendicular to the centerline, as demonstrated in Fig. 3a. In these planes, the field variables can be defined in an arbitrary point by interpolation from the global CFD mesh points. These cross-sectional planes are defined using the tangential unit-vector $\textbf{t}_f$.

#### Velocity Decomposition

To clarify the definitions, the component showing in the direction of the vessel centerline is called the tangential component. In the local system, the axial component defines the axial flow direction. The plane perpendicular to the tangential unit vector is the cross-section, and the velocity is the secondary component. The secondary component is further divided into the circumferential and radial components, respectively, representing local cylindrical coordinates (see 3b). In our application of the Frenet frames, the tangential unit vector was used to decompose the global velocity field into the axial component, then the secondary component, as follows. According to Eqs. 2, an arbitrary velocity vector can be decomposed into the axial flow $\mathbf {v_{ax}}$ and the secondary flow components $\mathbf {v_{sec}}$ defined using the tangential part of the Frenet unit vectors $\mathbf {t_f(p)}$ in the corresponding *p* centerline point. Then, by the second part in Eq. 2, the secondary flow vector $\mathbf {v_{sec}}$ is calculated by subtracting the axial flow vector $\mathbf {v_{ax}}$ from the velocity vector $\textbf{v}$.

2 $$\begin{aligned} \mathbf {v_{ax}} = \big (\textbf{v} \cdot \mathbf {t_f(p)}\big ) \mathbf {t_f(p)}, \; \mathbf {v_{sec}} = \textbf{v} - \mathbf {v_{ax}} \end{aligned}$$

The secondary flow components can be further separated into the radial and circumferential components by a local coordinate system transformation by switching to a polar representation around the centerline point ($\textbf{p}$) lying in the given cross-section. To calculate these components, first, a radial unit vector $\mathbf {r_{rad}}$ is defined by Eq. 3 between an arbitrary point ($\textbf{r}$) on the plane and ($\textbf{p}$). Then, using Eqs. 4, the secondary velocity vector can be decomposed into radial $\mathbf {v_{rad}}$ and circumferential $\mathbf {v_{cir}}$ components, respectively.

3 $$\begin{aligned} \mathbf {r_{rad}}&= \frac{\textbf{r}-\textbf{p}}{\mid \textbf{r}-\textbf{p} \mid } \end{aligned}$$

4 $$\begin{aligned} \mathbf {v_{rad}}&= \big (\mathbf {v_{sec}} \cdot \mathbf {r_{rad}}\big ) \mathbf {r_{rad}}, \; \mathbf {v_{cir}} = \mathbf {v_{sec}} - \mathbf {v_{rad}} \end{aligned}$$

Finally, the slices are transformed to the origin of the global coordinate system, offset, and then rotated to match the Frenet-frame unit vectors with the global axis directions ($\mathbf {n\rightarrow x,b\rightarrow y,t\rightarrow z}$), to express it in an illustrative way: the vessel is "straightened" along the global x-axis [34].

After transforming the slices, a region of interest (ROI) zone was defined. The ROI is defined by two assumptions. First, only the near-wall region affecting the innermost layer of the vessel (endothelium) is analyzed. The near wall region corresponds to the outer region defined by the radial coordinate of a unit parabolic profile at 50% of its peak. Second, we are interested in the difference between the cross-sectional region in which the *future* aneurysm lies and the opposite non-aneurysmal region (as depicted in Fig. 3 in panel c). After transforming the cross-sectional coordinates to the global coordinate system, the orientation for each aneurysm can be categorized into three groups, as shown in Fig. 3c according to the Frenet normal unit vector. The corresponding orientations are either when the aneurysm is leaning outside the bend (out) facing upward (top) or in a tilted fashion between the two directions. The angle of the aneurysmal segment is defined by $150^{\circ }$ since this angle range encompasses the aneurysm neck for all cases. Hence, each patient, after classification, was evaluated accordingly.

The calculated flow components are averaged in these zones along the arterial section. These averaged vector components (axial, radial, circumferential) provide a basis for understanding the influence of geometry and morphology on the flow structure.

### Further Data Reduction and Statistical Analysis

For statistical analysis, further data reduction was needed. To accomplish this task, an objective method was used to objectively separate the ICA into subsequent branch sections along the centerline.

**Fig. 4:**
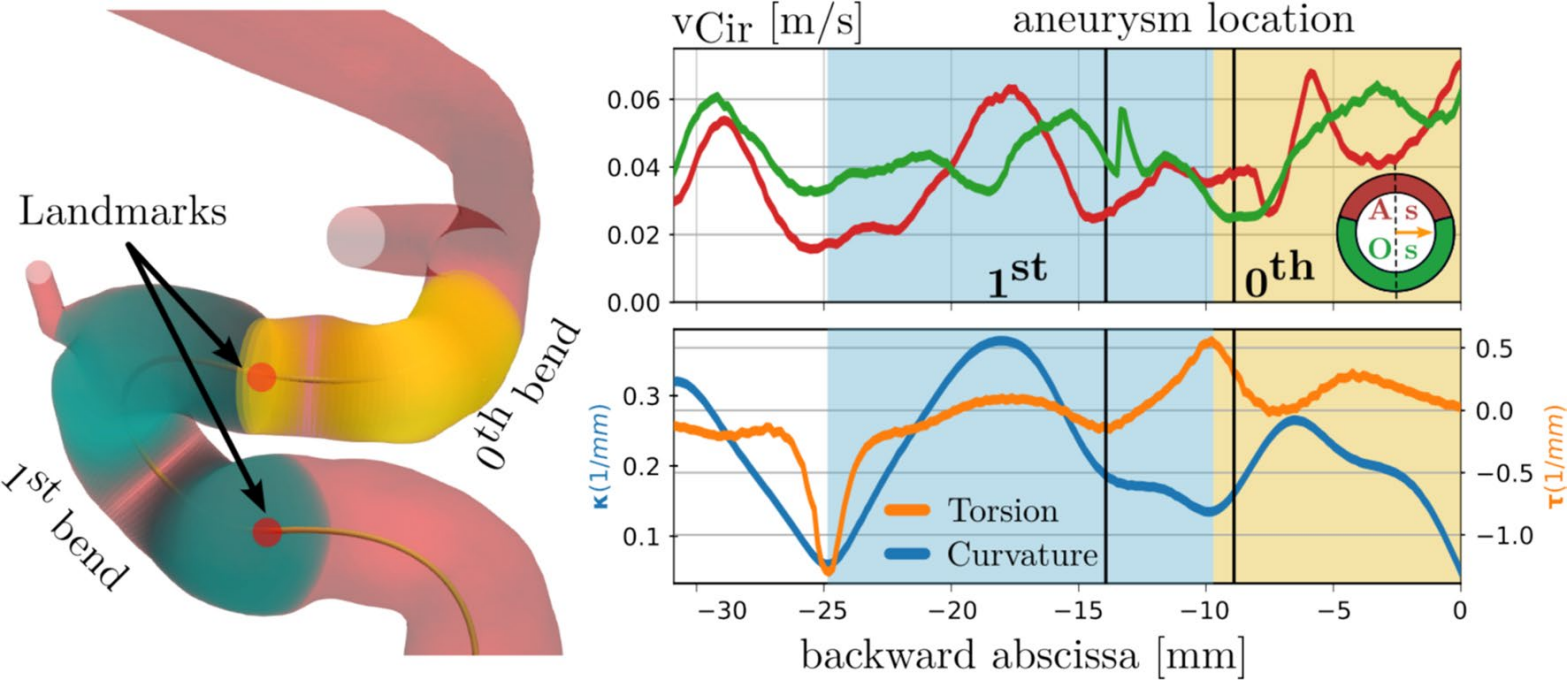
Landmarking procedure and subdivision of the flow field. Most of the aneurysms are located in the 1st bend.

Two methods exist in the literature for landmarking the ICA [28, 35]. Both methods are implemented in the MorhpMan Python toolbox [29], but since the centerline in this study is defined the same way as in [28], their method was chosen for the task.

The algorithm is based on identifying torsional and curvature peaks along the centerline. By definition, a branch section (an arterial bend) is a curvature peak surrounded by two torsional (distal and proximal) peaks. The borders are the landmark points that separate the centerline into subsequent bends, starting from the most proximal point of the centerline. The cross-sections can be grouped into corresponding branch sections using the landmarks along the centerline. Figure 4 shows the visualization of the procedure. The red spheres on the left depict the landmark points, which can be used to define sections. The resulting subdivision is marked on the corresponding circumferential velocity and centerline morphology diagram on the right to exemplify the next step. The values in the cross-sections corresponding to a given branch section are averaged to get a sectional mean of the velocity components and centerline morphological properties.

These sectional mean values were grouped into aneurysmal sections (A-bend) and other sections (O-bend) where there were no aneurysms and for the velocity components further into aneurysmal (A-side) and the opposing (O-side) zones according to the ROI definition explained in the previous section. Mean and standard deviations were calculated for all groups. The Welch T-test was used to quantify the significance of the difference between the two mean values as the variances were assumed unequal and the sample sizes were different. Finally, box-plot analysis was used to visualize the results.

## Results

The analysis of each case is visualized with the previously described composite diagram with the backward abscissa on the common x-axis. The first row describes the maximum inscribed sphere radius (MISR), and the following rows describe the velocity components in the following order: the axial $v_{axis}$, the circumferential $v_{cir}$, and the radial $v_{rad}$ velocities. The last row depicts the morphological properties of the centerline, the curvature (with the blue curve), and the torsion (with the orange curve). The green shaded region shows the site of the aneurysm based on the extent of the aneurysm neck. The diagrams for all cases can be found in the Appendix section. Here, only elucidative cases will be presented to understand the observations from the studied population.

### Flow Analysis Along the Centerline

**Fig. 5:**
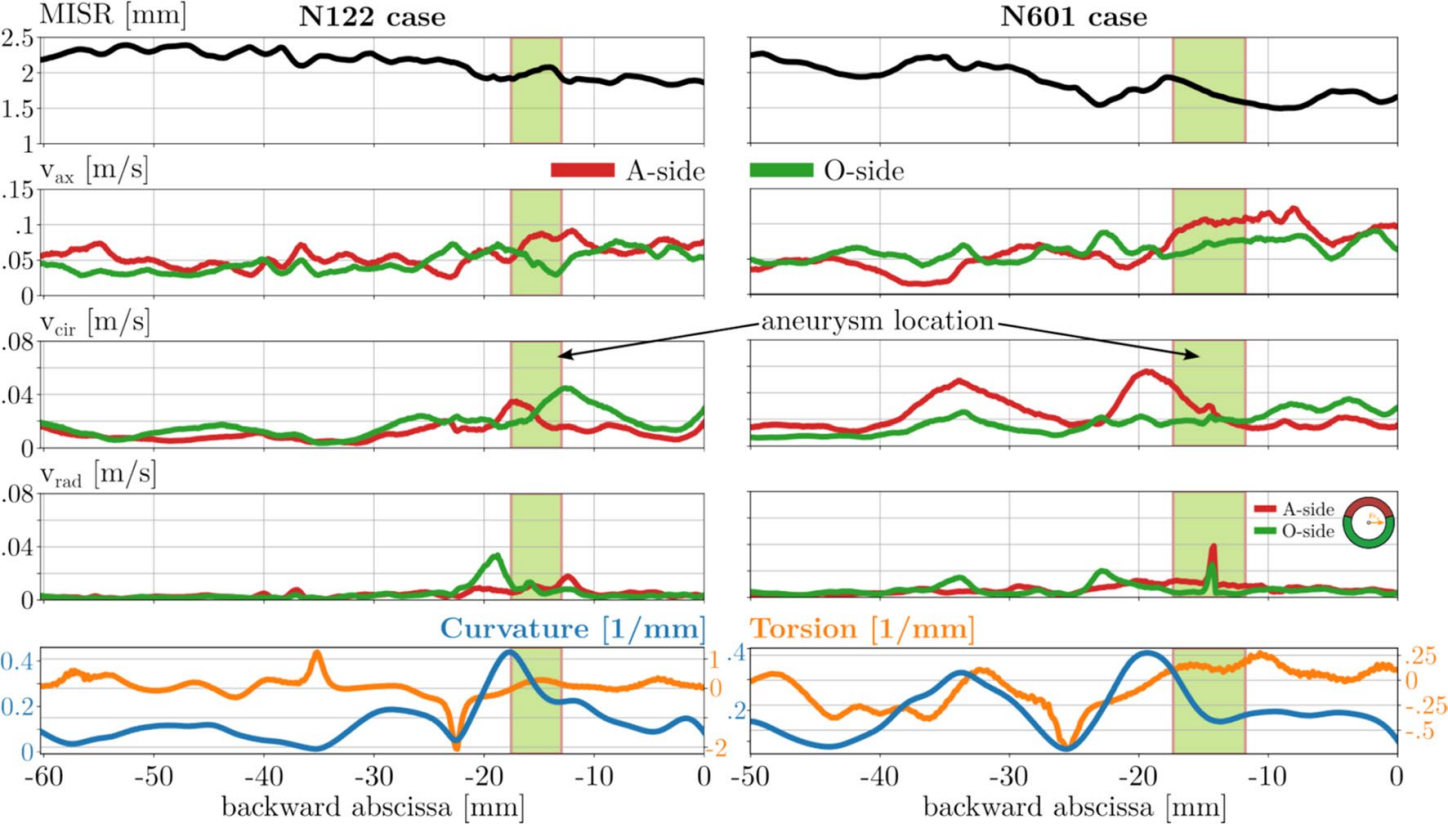
Composite diagrams of two example cases representing the expected cases. Left: N122 case. Right: N601 case.

After reviewing the results, 2/3 of the cases were similar in key features. Two cases are shown in Fig. 5 as examples to represent these key features. The original hypothesis claims that an intensive secondary flow emerges at the aneurysmal location. These thesis-supporting cases provide additional insight into how the morphological properties affect the velocity field. The following observations can be made about these cases: First, the aneurysmal formation site is in close proximity to the curvature peak point after the so-called clinoid bone, which is the usual formation site of parophtalmic aneurysms. Second, increased circumferential and radial flow activity is present in this neighborhood.

**Fig. 6:**
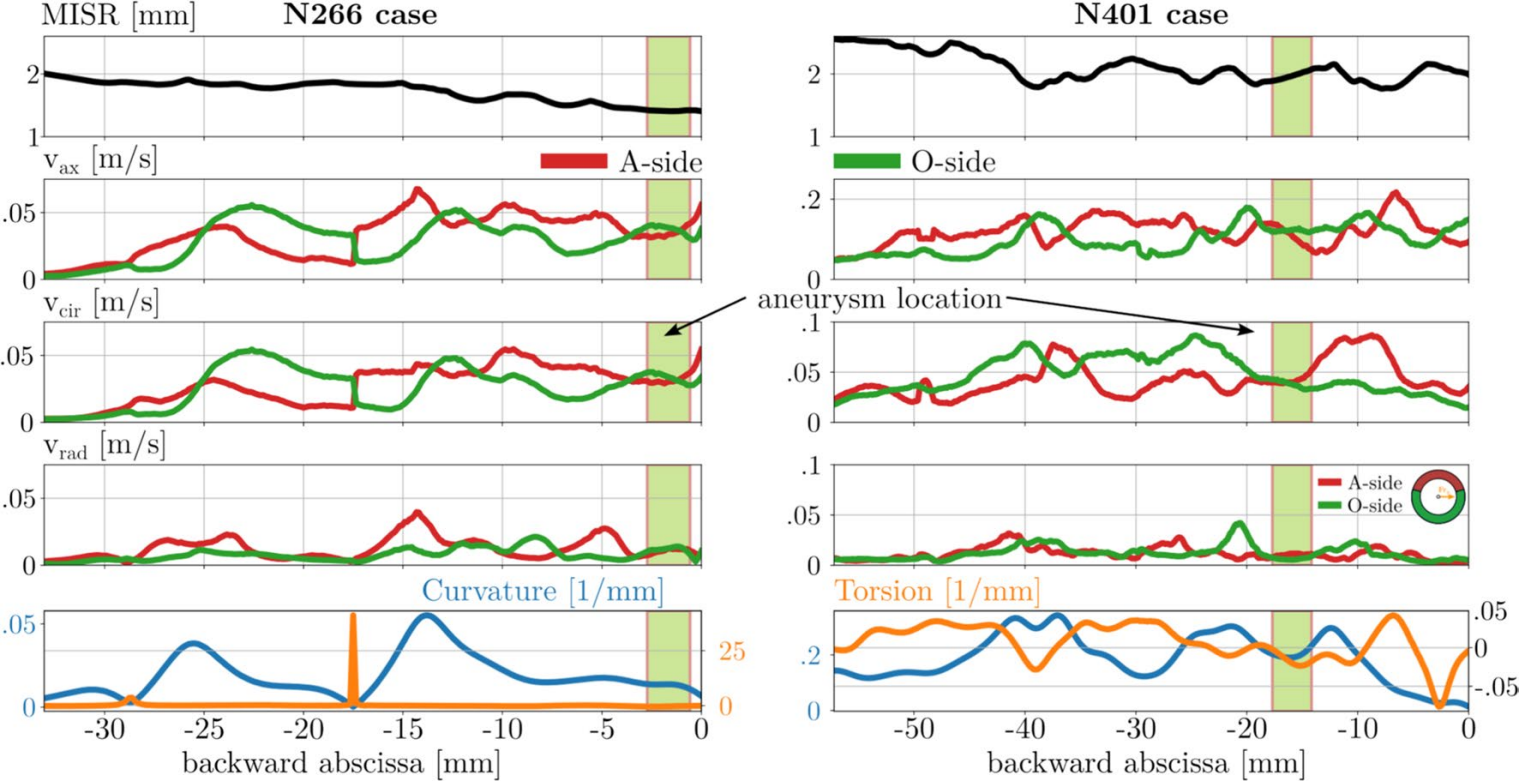
Composite diagrams of two example cases representing the unusual cases. Left: N266 case presenting a large torsional value. Right: N401 case with unusual MISR and small curvature variation.

About 1/3 of the cases do not fit in the above-described theory, though substantial similarities between the flow and morphology features can be found. In several cases, a small but not insignificant side branch of the ophthalmic artery is present at the anticipated site. In other cases, vessel radii or curvature variation along the artery is more complex, or an exceptionally high torsion is present (like the N266 case in Fig. 6) before the branch section with the future aneurysm.

### Branch Sectional Statistics

The previous section presented a qualitative overview of the flow field analysis. However, a statistical evaluation is required to interpret the relationship between the geometry of the branch sections and the emerging flow field. As mentioned before, the branch sections were defined based on the landmarking of the ICA centerline. On average, three subsequent branch sections could be identified for all thirty cases. In each branch section, the mean values were calculated and grouped according to the criteria mentioned in the methods section.

The highest occurring quantity value for a given case was used to normalize the velocity components (n-vec, n-ax, n-cir, n-rad) and morphological properties to get a comparative basis for the studied population. The normalization step was necessary due to the different vessel sizes. Larger vessel diameters entail higher velocities in all components, which would impose dimensional bias into our data. Several classifications can be made here, but the most important one is to compare the hosting branch section (A-bend) with the other (O-bend) section. In the case of the velocity components, another relevant classification can be made by arranging the values further into the aneurysmal (A-side) and other side (O-side) groups.

**Fig. 7:**
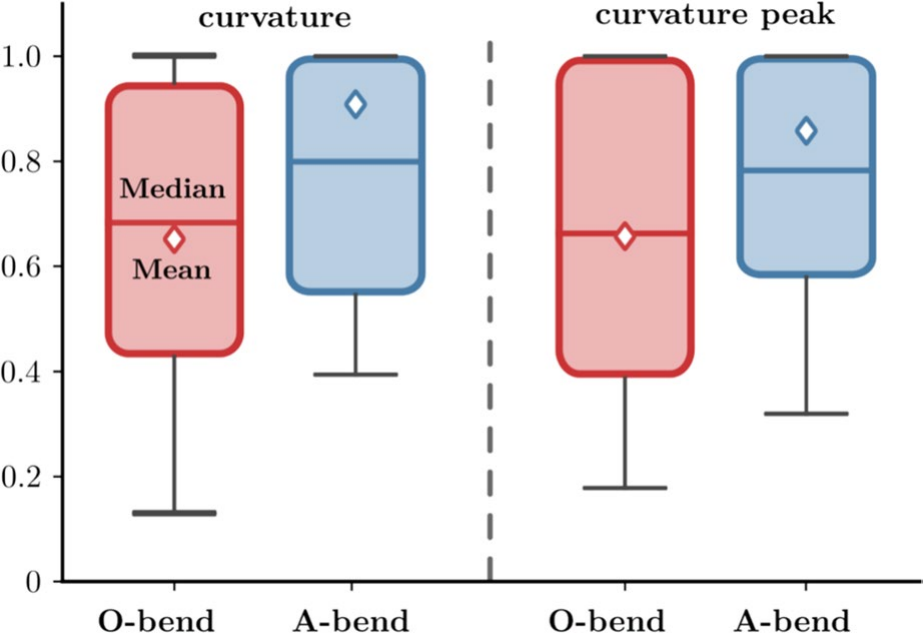
Box plots of normalized mean curvature and curvature mean values classified into aneurysm bend (A-bend) and other bends (O-bend) groups. The red/blue line and diamond depict the median and the mean values, respectively.

Figure 7 displays the box plots for the normalized curvature and curvature peaks. Blue and red boxes correspond to the aneurysm bend (A-bend) and other bends (O-bend) groups. The red/blue line and diamond depict the median and the mean values, respectively. The box plots of both morphological quantities demonstrate that the case-relative mean curvature and curvature peak are higher in the hosting bends. Table 2 summarizes the statistical data. The last two rows demonstrate the difference between the averages. The Welch T-test was used to quantify the significance of the difference between the two mean values as the variances were assumed unequal and the sample sizes were different. Torsional values (mean torsion, proximal and distal torsions) were also evaluated, but only insignificant and minor differences were obtained. Significant differences in means were obtained for both the normalized mean curvature and curvature peak. The result indicates that an aneurysm will likely form on the arterial bend with a higher relative curvature for a given case.

**Table 2:** Summary of the normalized sectional mean velocity components and their standard deviations comparing the averages of the host bend and other bends

Quantity	A-bend $\rightarrow$ A-side(mean±SD)	O-bend $\rightarrow$A-side (mean±SD)	p-value
n-vec	0.834 ± 0.153	0.754 ± 179	0.0274*
n-ax	0.763 ± 0.186	0.699 ± 0.215	0.1471
n-cir	0.774 ± 0.217	0.622 ± 0.234	0.0029*
n-rad	0.785 ± 0.226	0.622 ± 0.234	0.0057*

	HostBend	OtherBend	
nKappa	0.781 ± 0.232	0.661 ± 0.284	0.0332*
nKappaMax	0.798 ± 0.228	0.681 ± 0.268	0.0332*

Significance was calculated with the Welch T-test due to unequal variances. Significant results are depicted with a *sign if the p-value is lower than 0.05

**Fig. 8:**
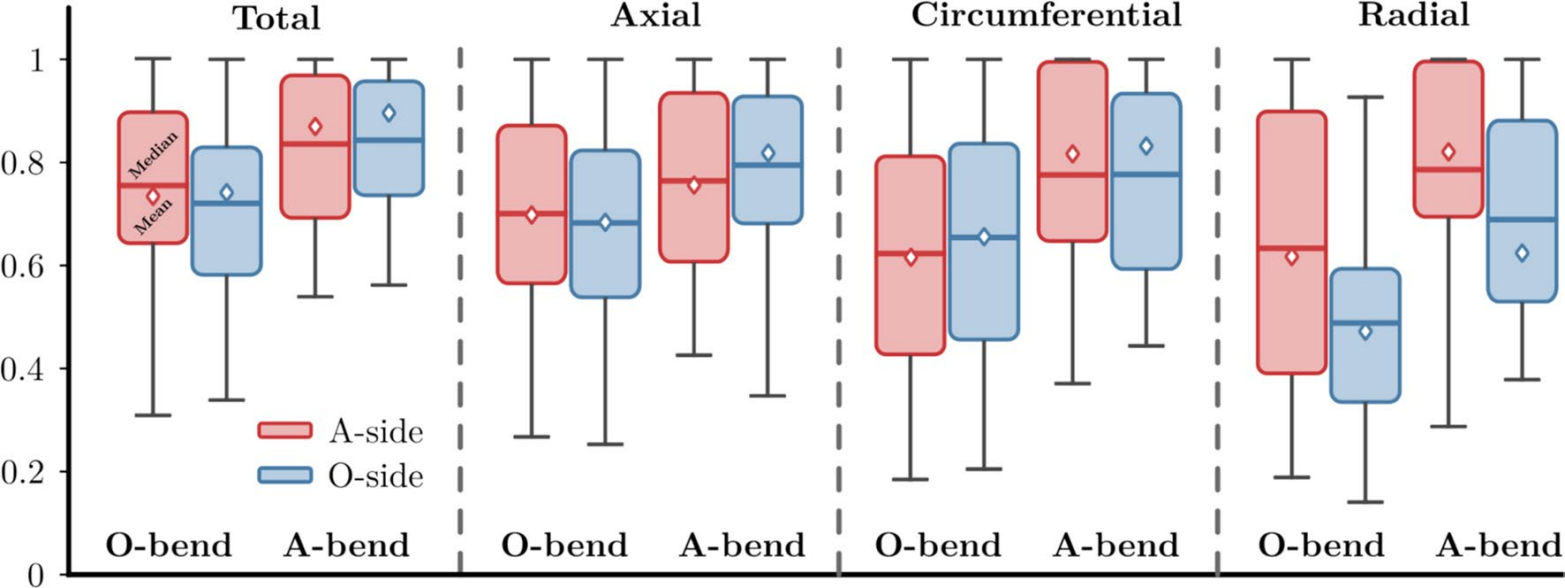
Box plots of normalized mean velocity components classified into the aneurysm (A-bend) and other bend (O-bend) groups and subclassifying into groups corresponding to aneurysmal (A-side) and other side (O-Side) groups. The red/blue line and diamond depict the median and the mean values, respectively.

Figure 8 displays the box plot evaluations of the normalized velocity components. Compared to the morphological properties, the velocities can be further subclassified into groups corresponding to the aneurysmal (A-side) and other sides (O-side). However, looking at the box plots, it is apparent that with the exception of the normalized mean radial velocity, only an insignificant difference can be observed between the mean values of the *A-side* and *O-side*. The observation would indicate that the underlying fluid mechanic influence is not focally emerging but rather a property of the centerline morphology. On the other hand, this is not true for the radial velocities. In the current use of the evaluation framework, only vector magnitudes were analyzed, and information on the direction was not captured. This information could help in understanding the radial flow field in the future. Group mean values, standard deviations, and significance levels for comparing the means are plotted in Table 2. A p-value lower than 0.05 is considered significant. The velocity components between the aneurysmal and non-aneurysmal branch sections are compared using the *A-side* zone since an aneurysm is expected at the *hosting* side. Looking at Table 2, significant differences were found between the averages of the normalized velocity ($p=0.0274$), the circumferential ($p=0.0029$), and radial ($p=0.0057$) velocity components in aneurysm-hosting and non-aneurysm-hosting branch sections. However, the difference of means for the normalized axial component is insignificant ($p=0.1471$) between the aneurysmal branch section and the other section groups.

## Discussion

### Morphology and Flow

The hypothesis emerging from the pilot study was the emergence of intensified secondary flows at the aneurysm site. In this study, we identified typical flow and centerline features corresponding to the morphological features of the parent vessel and the later aneurysmal formation site. The secondary flow field was visualized in [3] as early as 2006 for the pre-aneurysmal flow field, but it was not discussed until later [4, 5], even though the effect of helical flows in other arteries are heavily studied [36–39].

It can be recognized how morphological features influence the flow field. With the exception of a few cases, the axial velocity is mainly influenced by the maximum inscribed sphere (MISR) variation along the centerline. It is not surprising at all, as it meets the expectations when the vessel constricts, the velocity increases, and when the vessel expands, the velocity decreases. Another interesting observation was the similarity between the curvature and the circumferential velocity component along the centerline. A possible explanation might be that the Dean number also increases as the curvature increases, implying an increased secondary flow activity. The underlying physics is more complicated due to the complex morphology properties of the vessel, and the scope of the present study was not to deeply understand the connection between these features. Still, it will be of great interest in future studies.

### Statistical Interpretation

The statistics revealed that the normalized total, circumferential, and radial flow components are significantly higher in the aneurysmal branch sections than in the non-hosting bends. At the same time, this is not true for the axial component. Furthermore, the case-relatively normalized mean and peak curvatures are significantly higher in the hosting bends, similar to other findings [11, 28]. The proposed pathomechanism might be the following. For a given case, the distal ICA leading towards the circle of Willis consists of several arterial bends. The occurrence of an aneurysm is more probable in the bend with a higher relative curvature.

The statistical interpretation may be as follows. The case-relative velocity in the hosting bend is higher than in other bends, yet this information does not necessarily mean higher secondary flows in the hosting bend. However, the fact that the case-relative axial flow is not significantly higher, while the radial and circumferential component is, implies that secondary flow features play a role in the development of sidewall intracranial aneurysms.

The biomechanical interpretation of these results is that amplifying secondary flow could emerge in the presence of higher relative curvature, generating high WSS regions whose direction is not aligned with the bulk flow. As observed in our pilot study [24], the secondary flows might exert a directionally time-varying load on the vessel wall, generating a fluctuating WSS field. The fluctuating load through mechanotransduction could trigger parts of the biochemical pathways in the cascade process of vessel-wall remodeling [22]. Here, a remark is warranted. The results described in this paper are just the first step to shed some light on this topic, as the transient nature of the flow field has not yet been analyzed.

### Limitations and Future Work

The main limitation of the present study is that control cases were not compared against the studied population. Very soon, medical images of a healthy population will be acquired and studied. Second, although objective, the virtual removal technique may not accurately represent the vascular morphology change during the growth of the aneurysm [40]. However, in the case of sidewall ICA aneurysms, the shape of the parent vessel is relatively unaffected by the formation [3]. The flow rate waveform is a statistical average of healthy subjects [30]. Patient-specific boundary conditions that reflect the different states of the person would be ideal to give the entire picture of the hemodynamic condition. In the near future, a formal uncertainty quantification will be carried out to assess the aforementioned aspects. Furthermore, the study only included ICA aneurysms located after the clinoid bend. The underlying mechanisms of the formation of bifurcation aneurysms might differ, but a detailed flow field evaluation to understand the induced WSS field should be analyzed. Last, the usual limitations of CFD modeling are mentioned. Namely, the blood was assumed to be Newtonian, and the vessel walls were rigid. These approximations are widely accepted in the literature.

## Conclusion

This study aimed at finding a link between the global geometrical features of the vessel and the emerging flow field on one side and the location of the aneurysm on the other. For this purpose, a common evaluation framework was developed to quantify the hemodynamic field in the same frame of reference as the arterial centerline and associated geometrical properties. The framework can be used and extended according to the need to analyze the cause-and-effect relationship between geometrical properties and the complex hemodynamic field. Thirty cases with aneurysms located mostly on the parophtalmic segment of the ICA were analyzed in the virtually reconstructed pre-aneurysmal state. Based on statistical analysis, we found that sidewall aneurysm formation on the ICA is more probable in those arterial bends where the highest case-specific curvature induces the highest case-specific secondary flow (circumferential and radial velocity components). The use of vector field decomposition into physiologically relevant projections along the centerline can quantify the unfavorable fluid dynamic loads and relate them to the biological sensing mechanisms of the vessel wall.

## Supplementary Information

Below is the link to the electronic supplementary material.

Supplementary file1 (PDF 13,190KB)
