# Supplementary material for: Amplification of Secondary Flow at the Initiation Site of Intracranial Sidewall Aneurysms
Source: Cardiovasc Eng Technol. 2025 Jan 27;16(3):259–71. doi: 10.1007/s13239-025-00771-4 (PMC12130061; doi:10.1007/s13239-025-00771-4)
Supplement: Supplementary file 1 — Supplementary file1 (PDF 13,190KB) [file 13239_2025_771_MOESM1_ESM.pdf]

Appendix: Composite diagrams

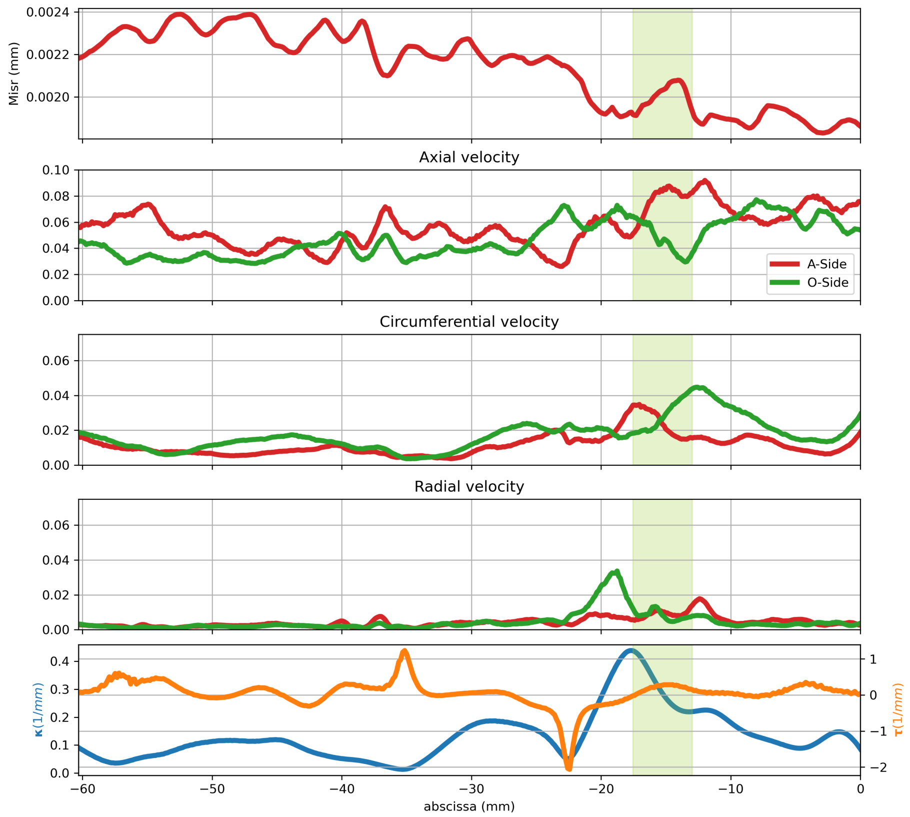

Figure A1: Composite diagrams of case N122.

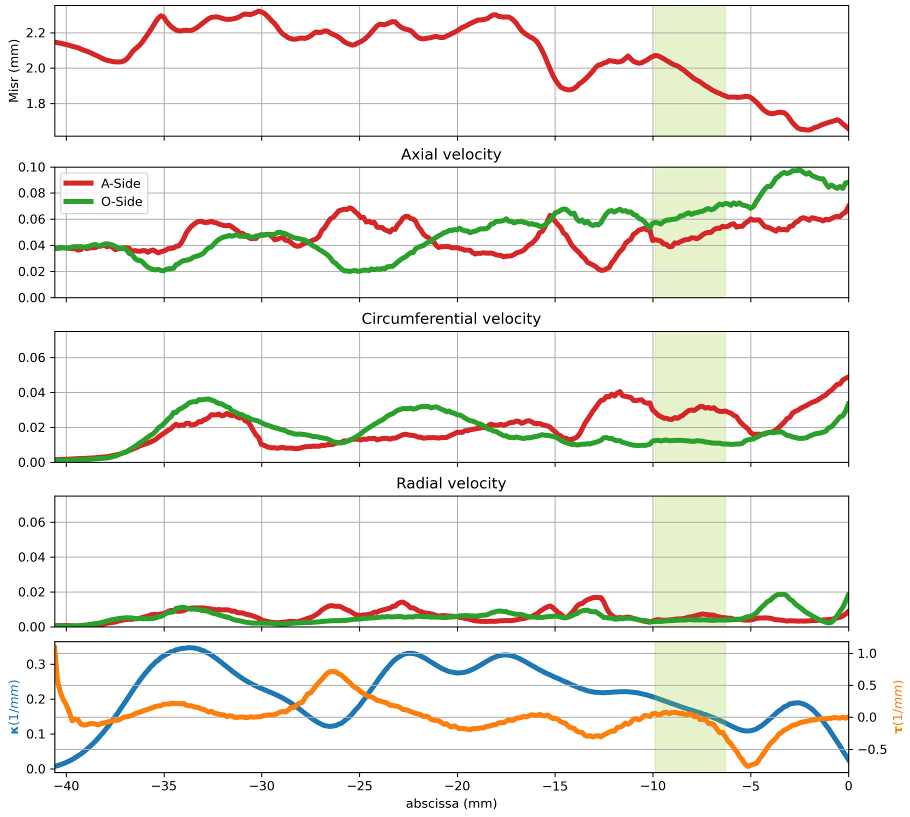

Figure A2: Composite diagrams of case N126.

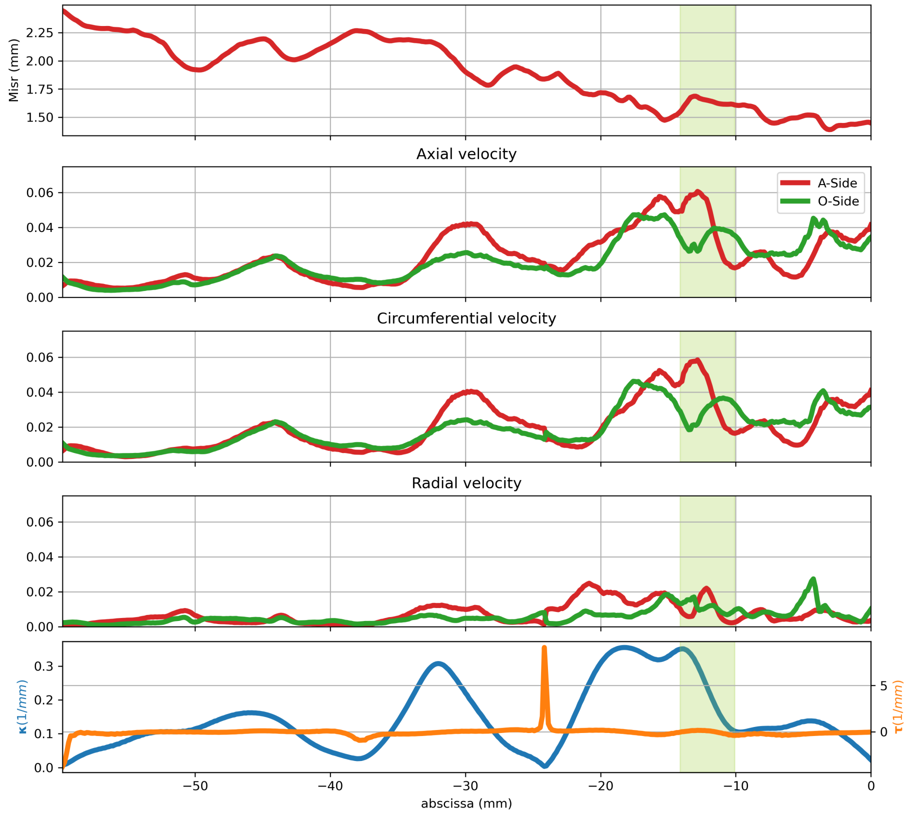

Figure A3: Composite diagrams of case N168.

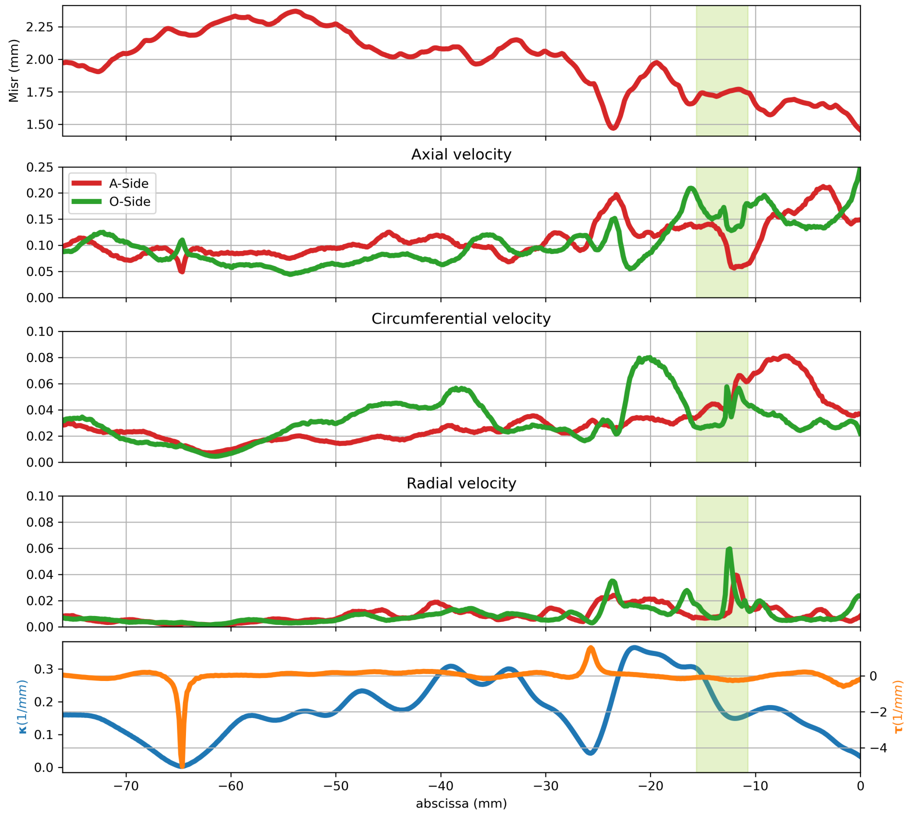

Figure A4: Composite diagrams of case N173.

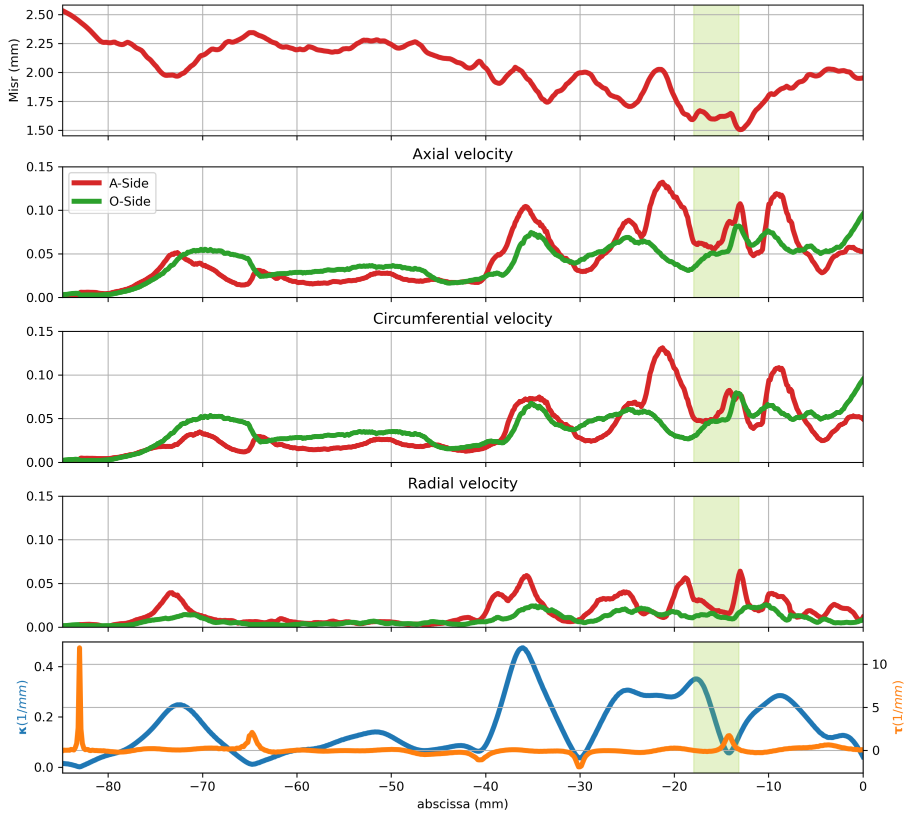

Figure A5: Composite diagrams of case N180.

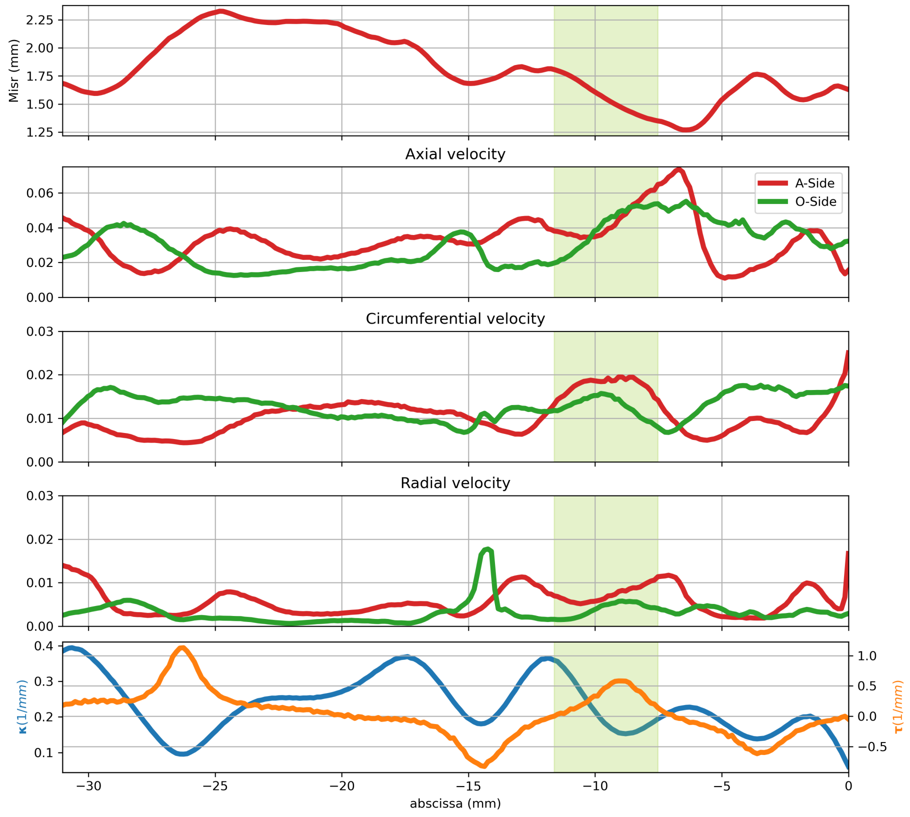

Figure A6: Composite diagrams of case N212.

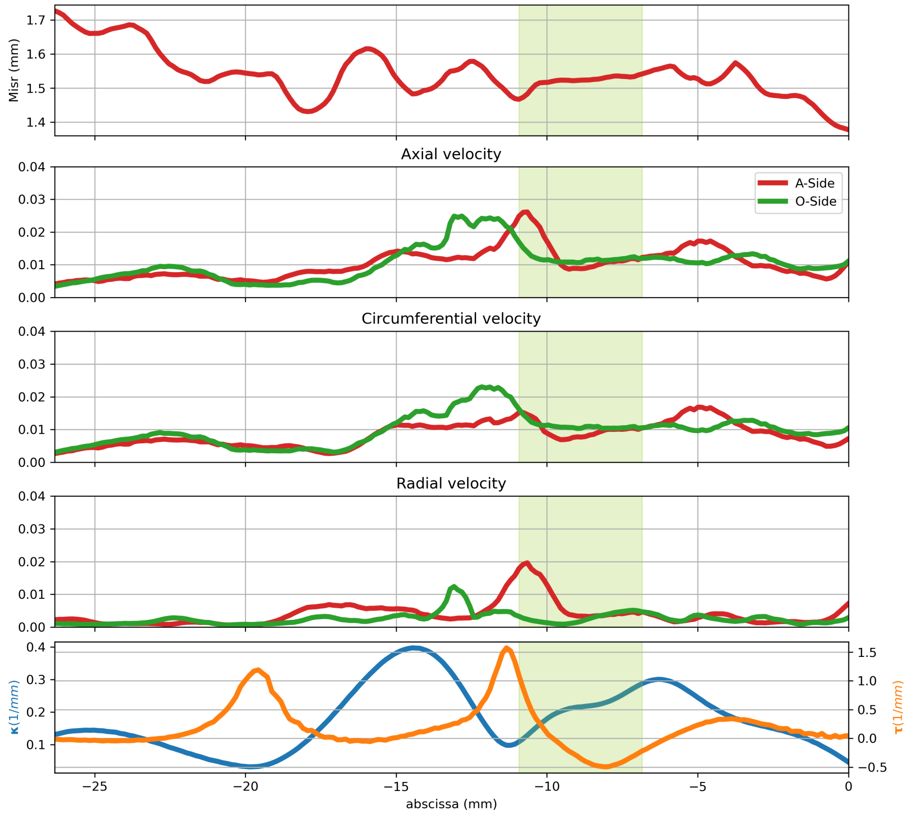

Figure A7: Composite diagrams of case N237.

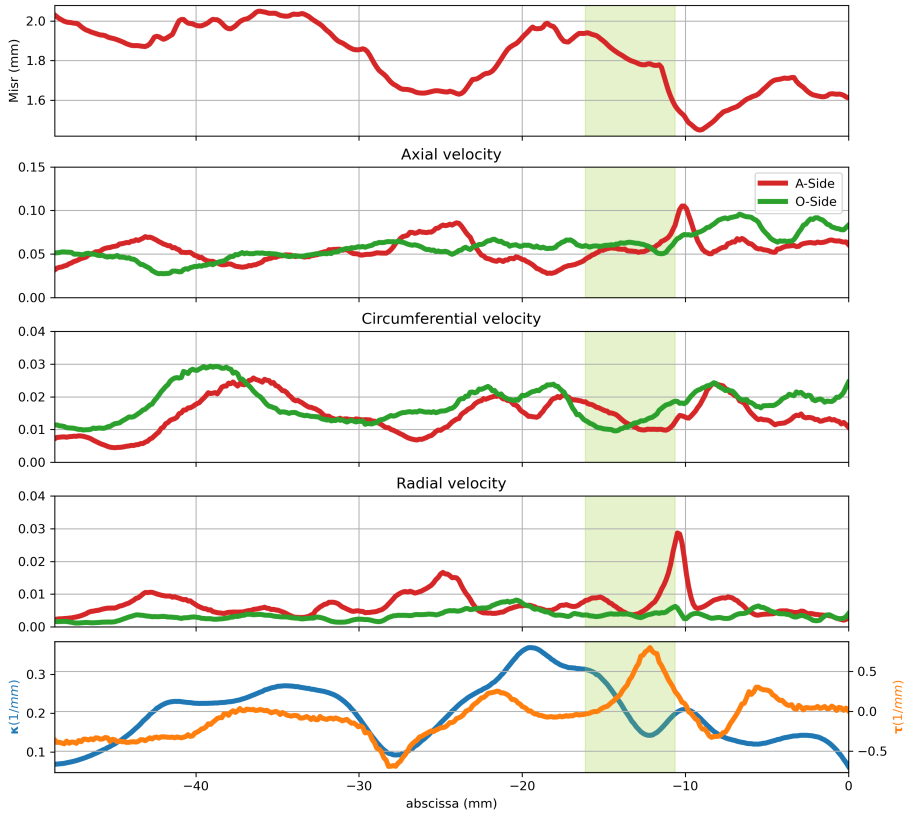

Figure A8: Composite diagrams of case N251

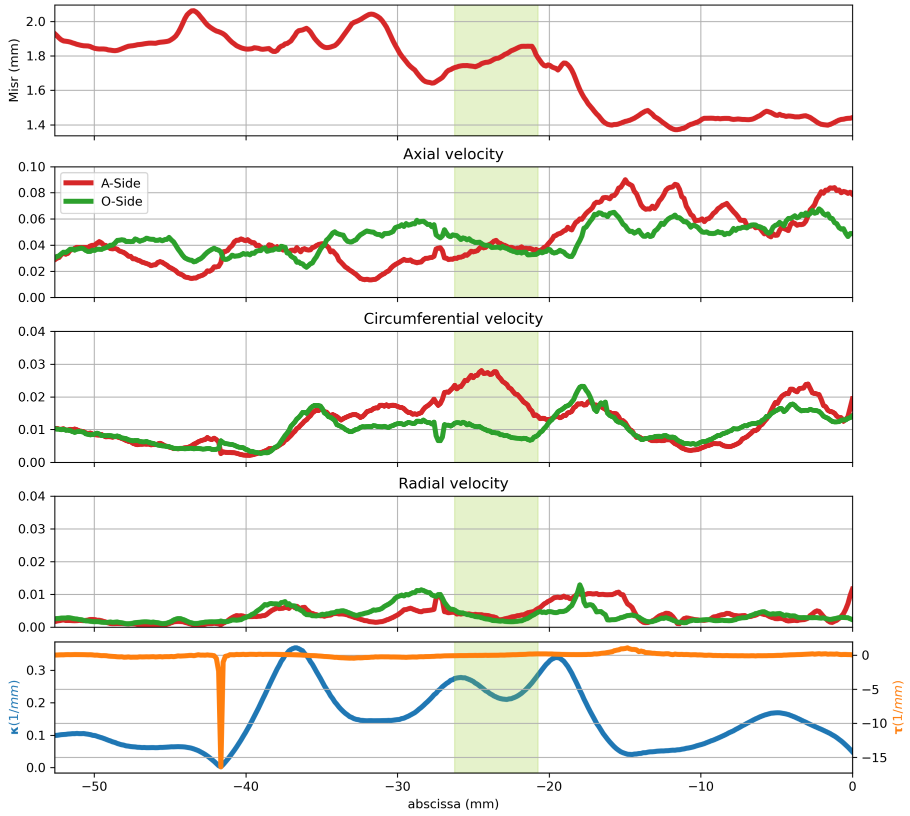

Figure A9: Composite diagrams of case N252.

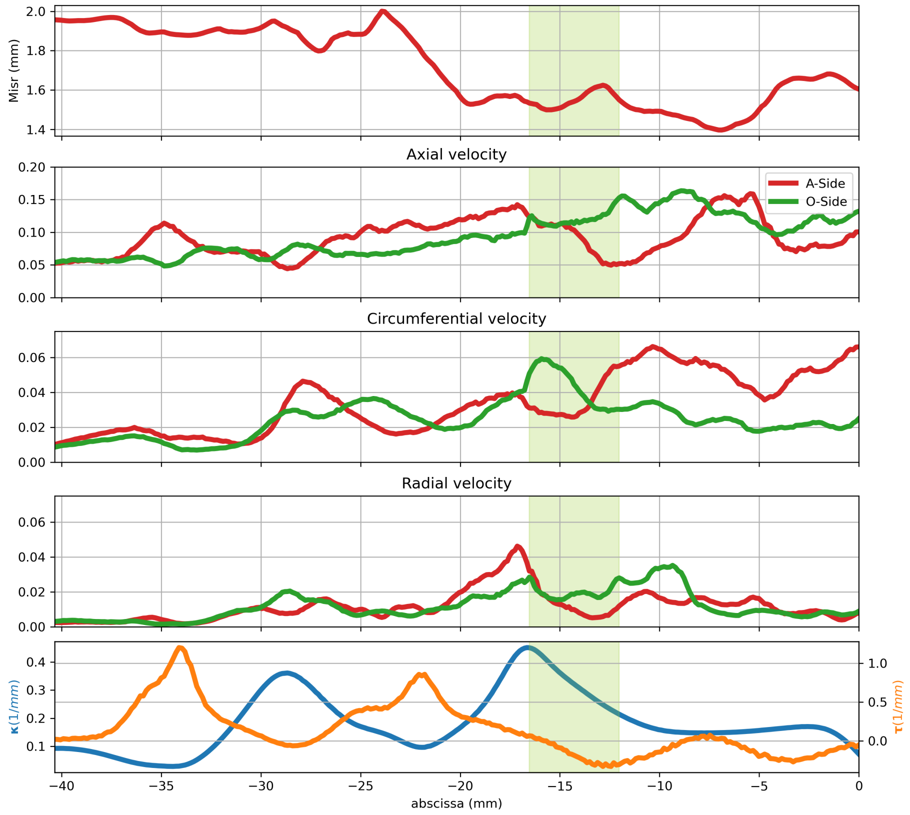

Figure A10: Composite diagrams of case N256.

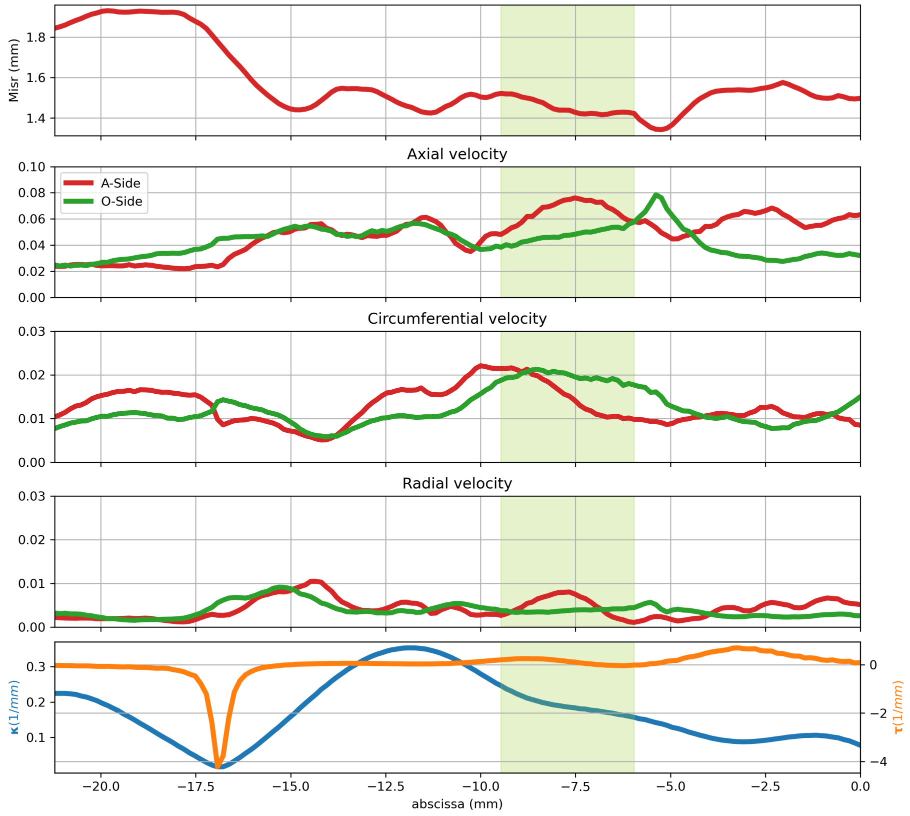

Figure A11: Composite diagrams of case N259.

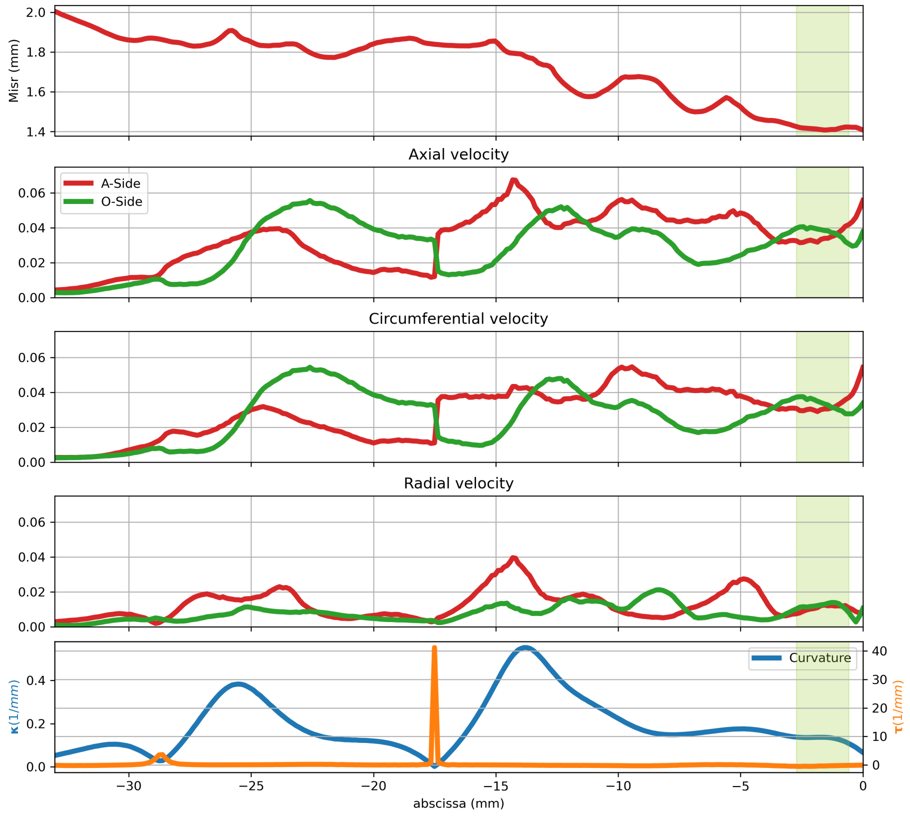

Figure A12: Composite diagrams of case N266.

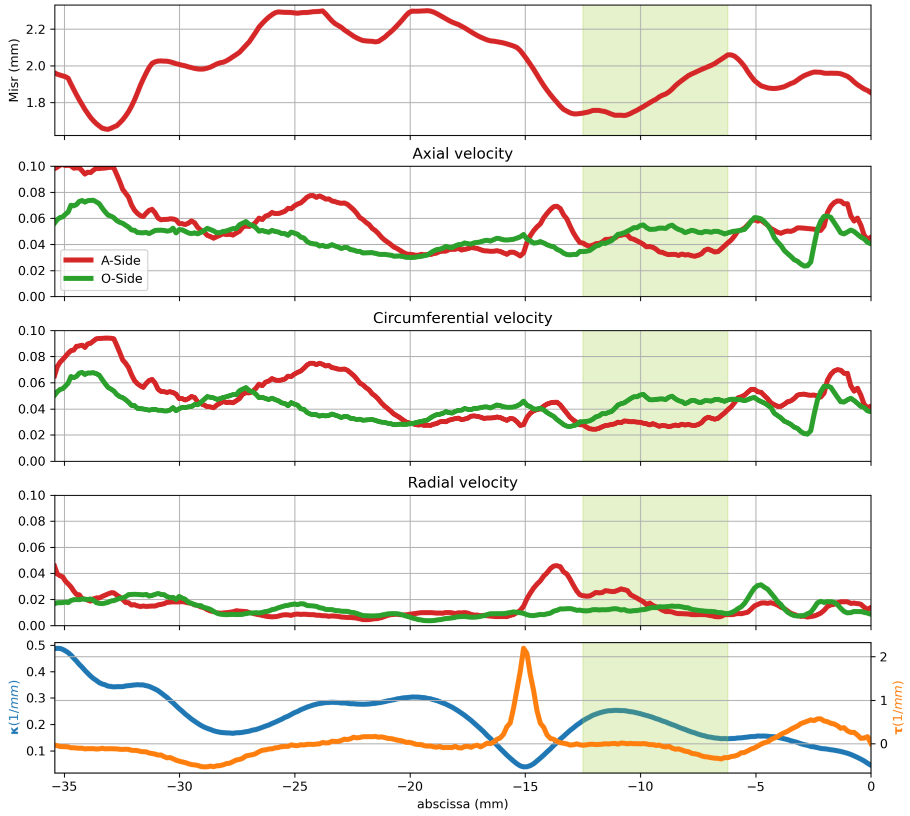

Figure A13: Composite diagrams of case N270.

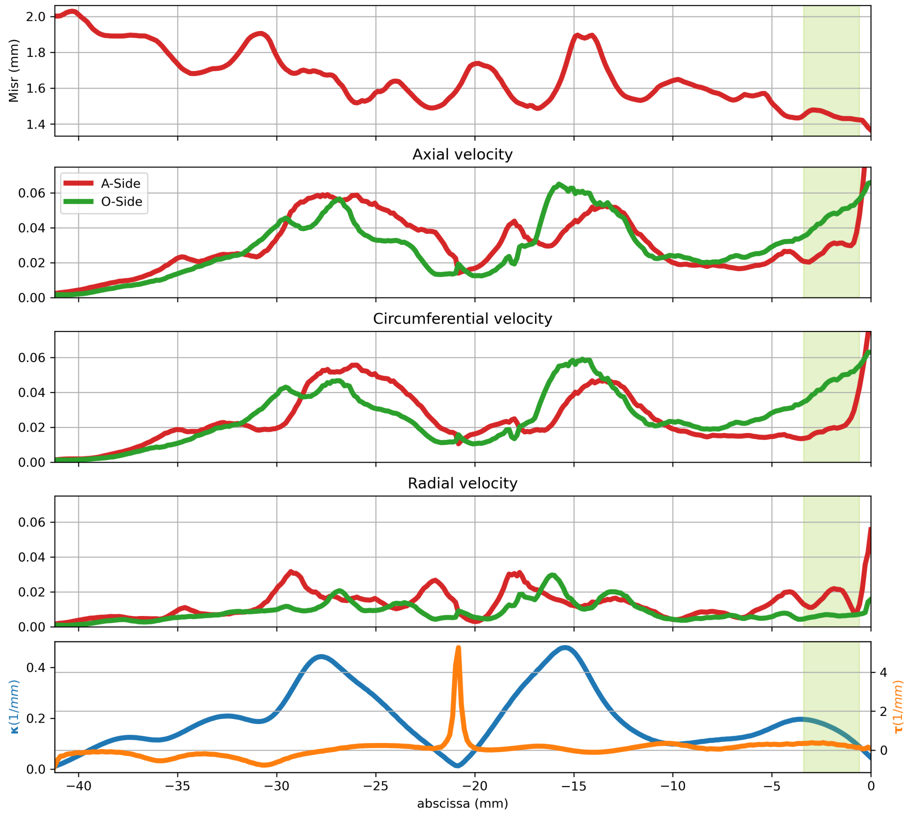

Figure A14: Composite diagrams of case N271.

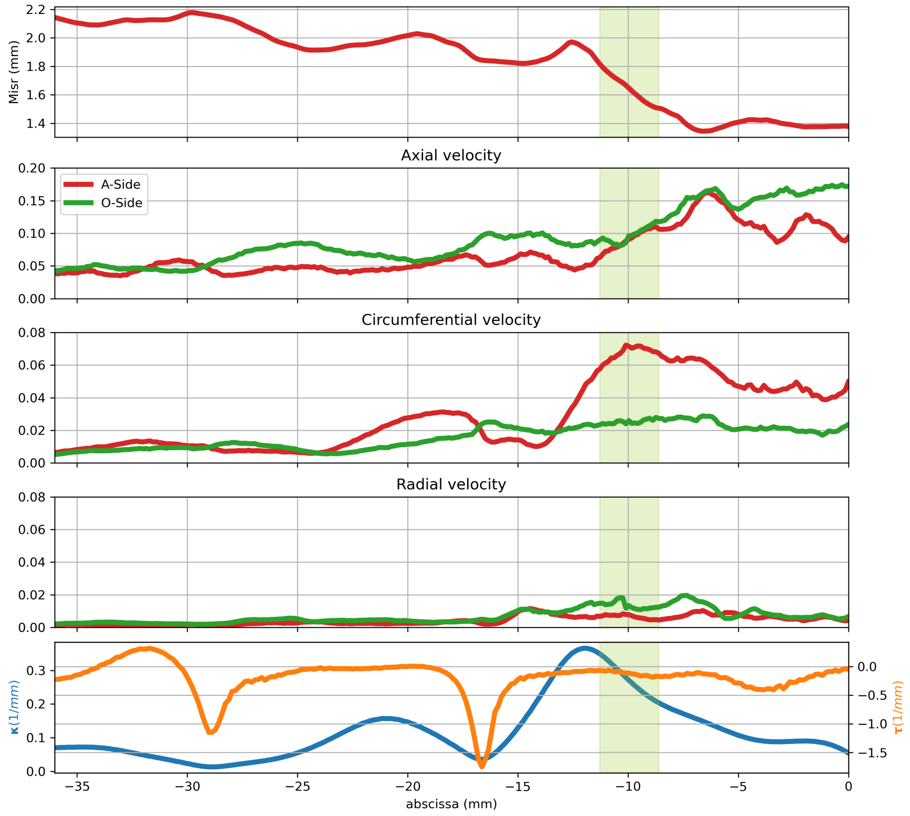

Figure A15: Composite diagrams of case N274.

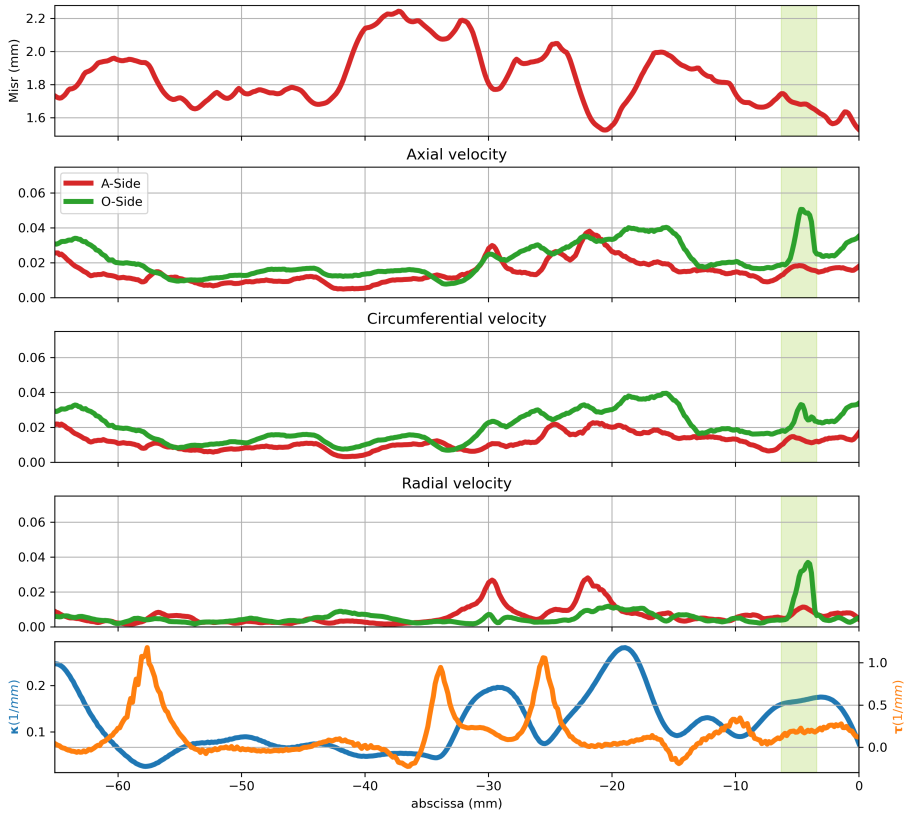

Figure A16: Composite diagrams of case N276.

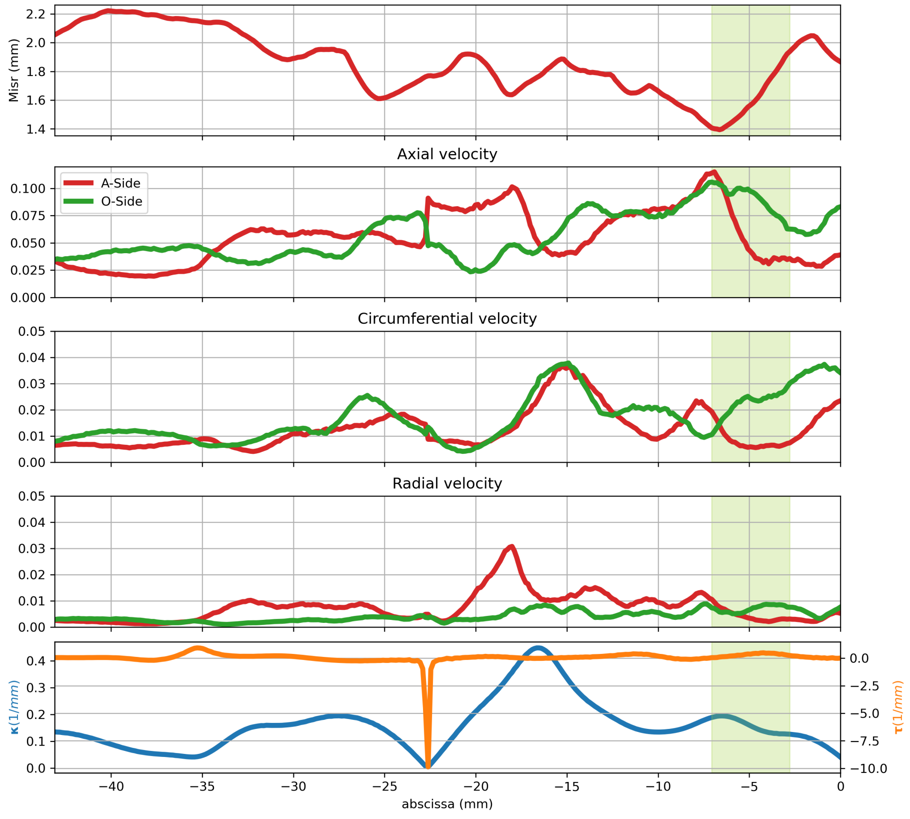

Figure A17: Composite diagrams of case N285.

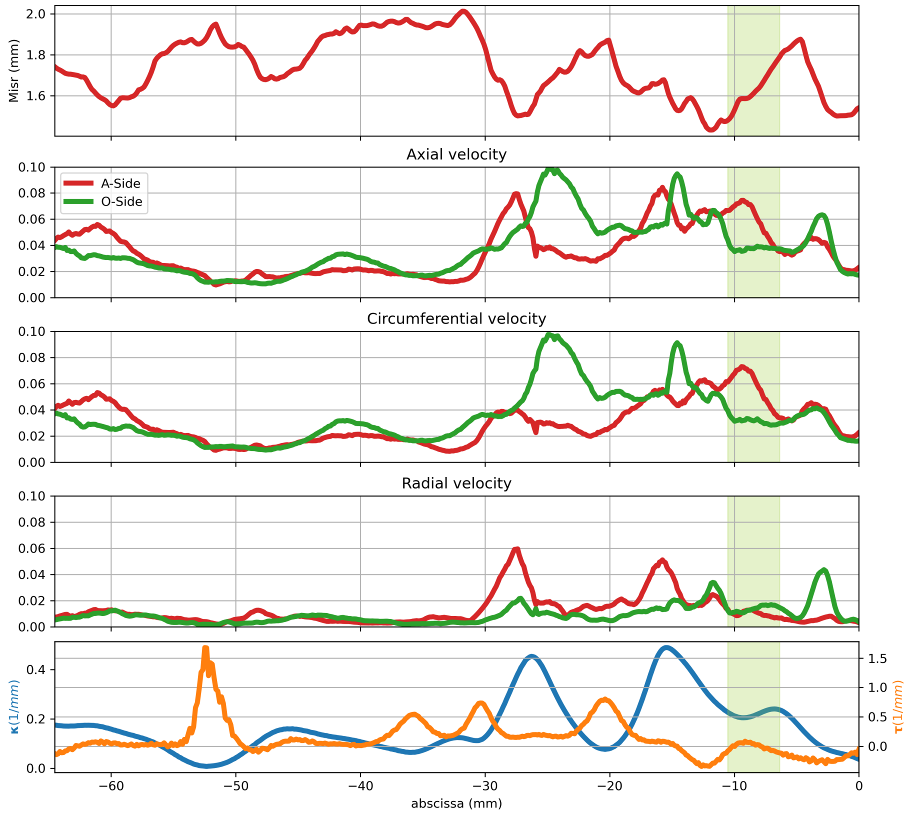

Figure A18: Composite diagrams of case N287.

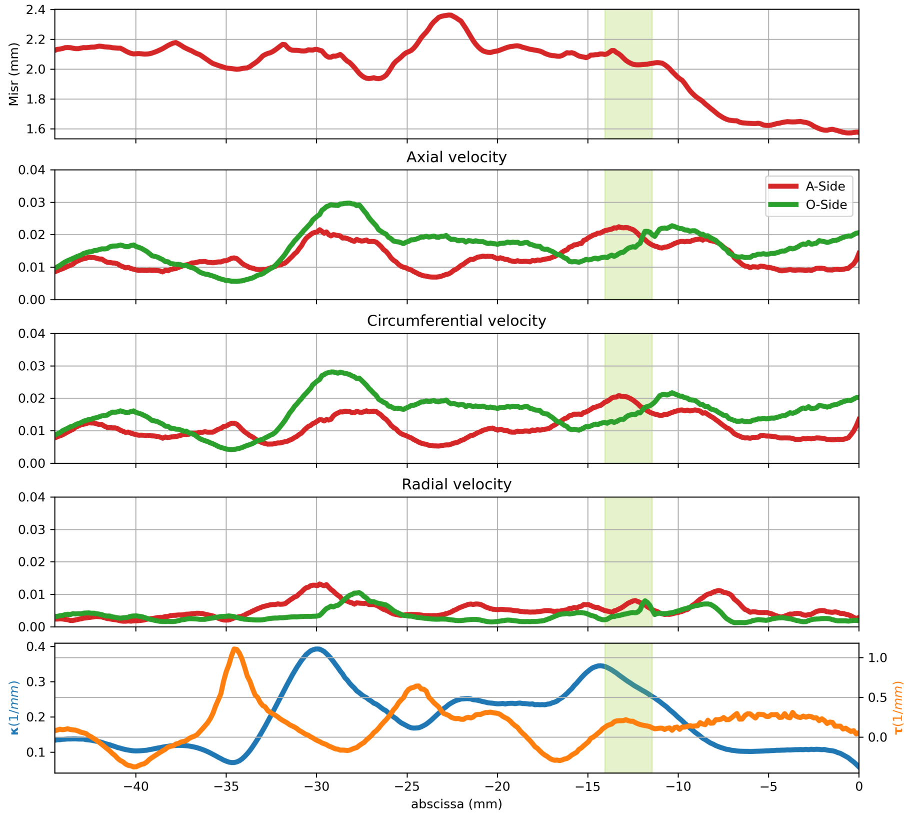

Figure A19: Composite diagrams of case N292.

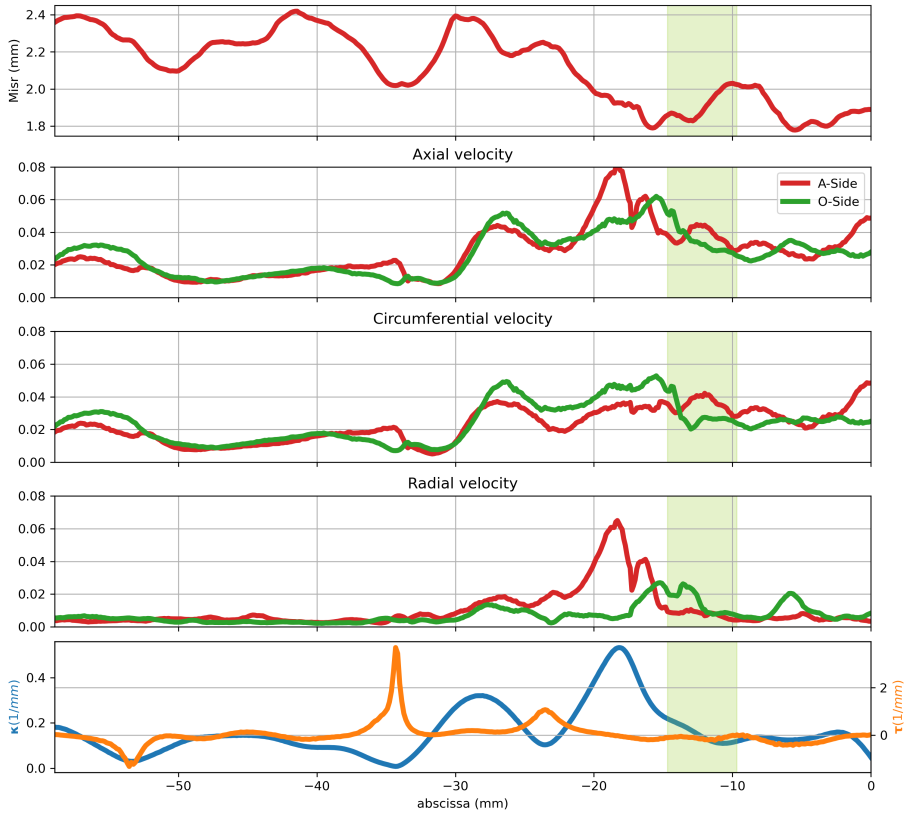

Figure A20: Composite diagrams of case N297.

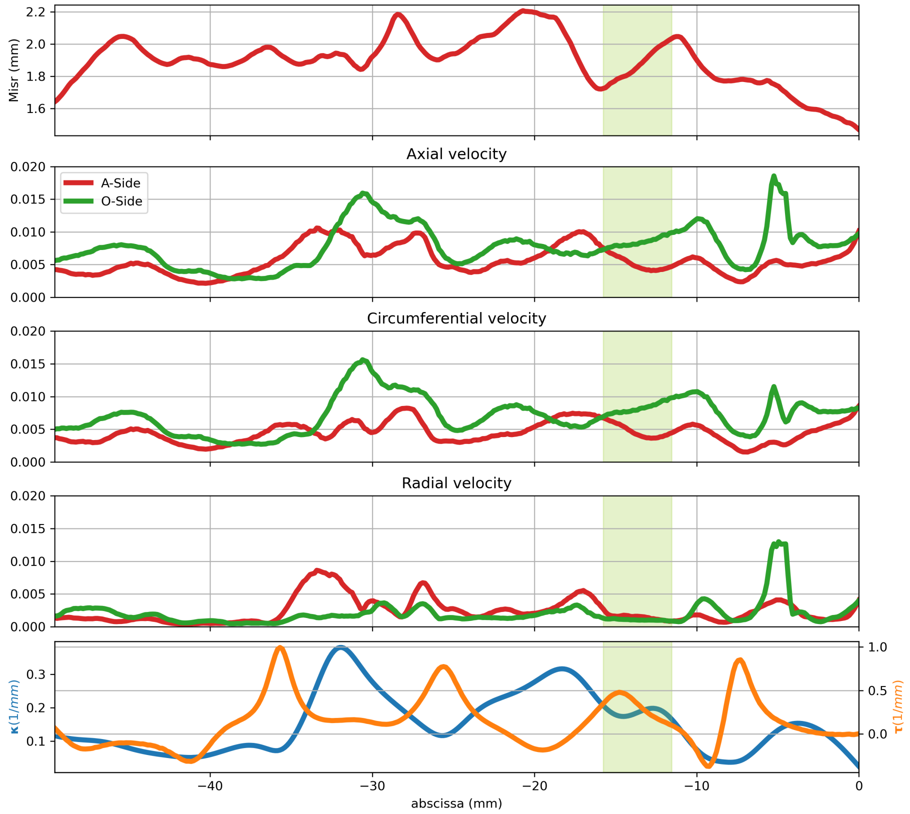

Figure A21: Composite diagrams of case 301.

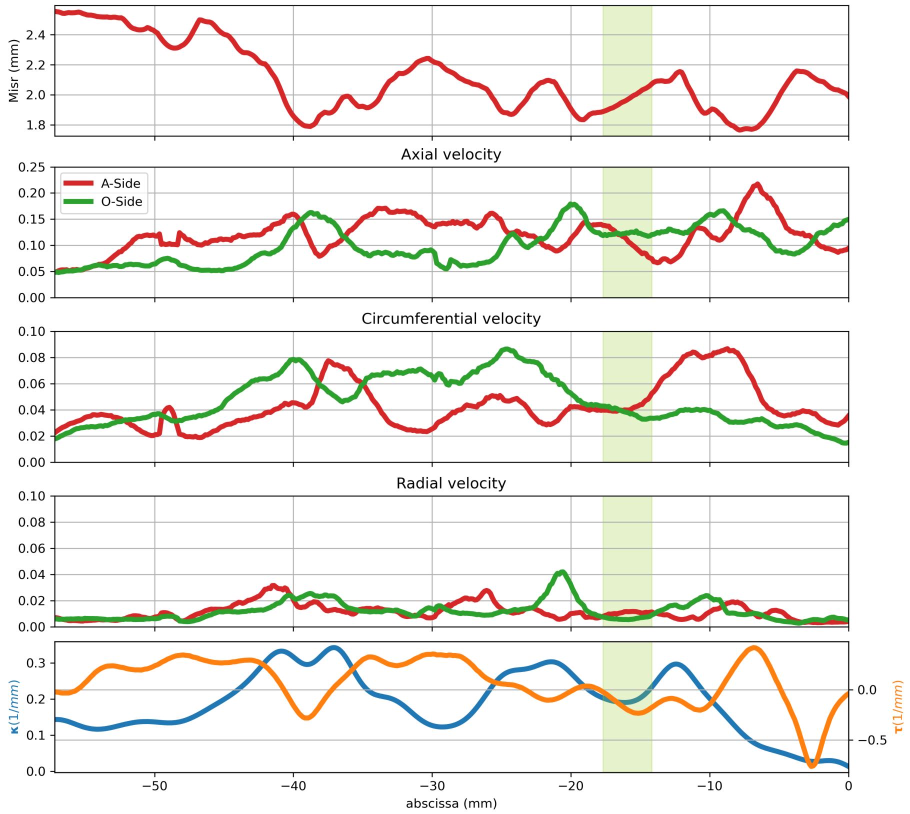

Figure A22: Composite diagrams of case N401.

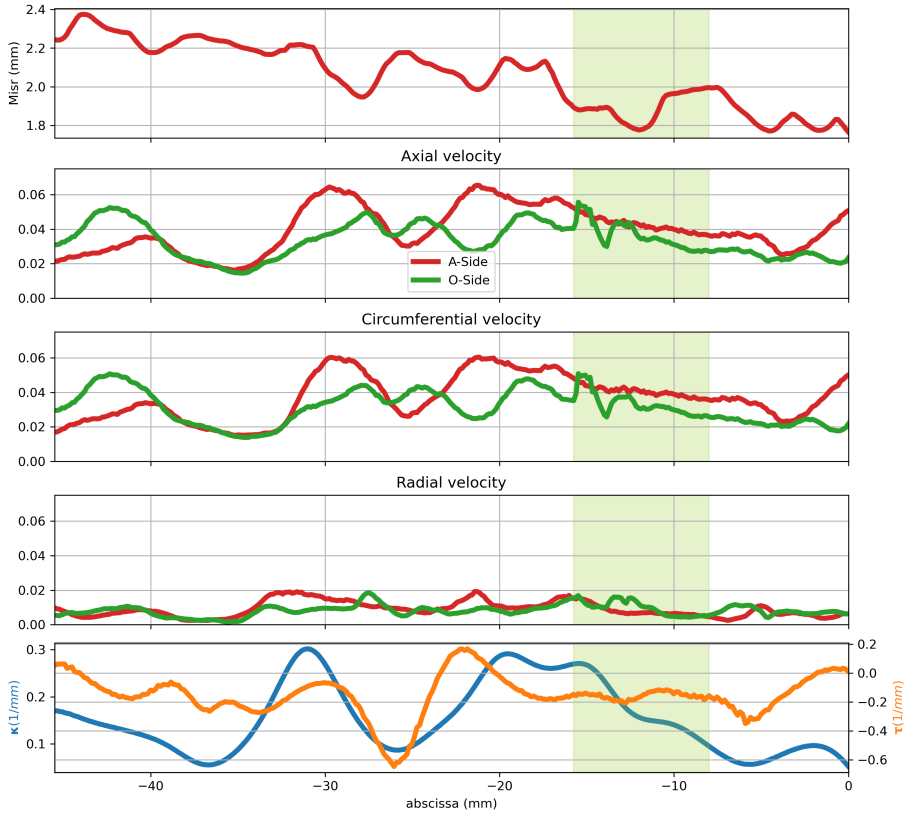

Figure A23: Composite diagrams of case N402.

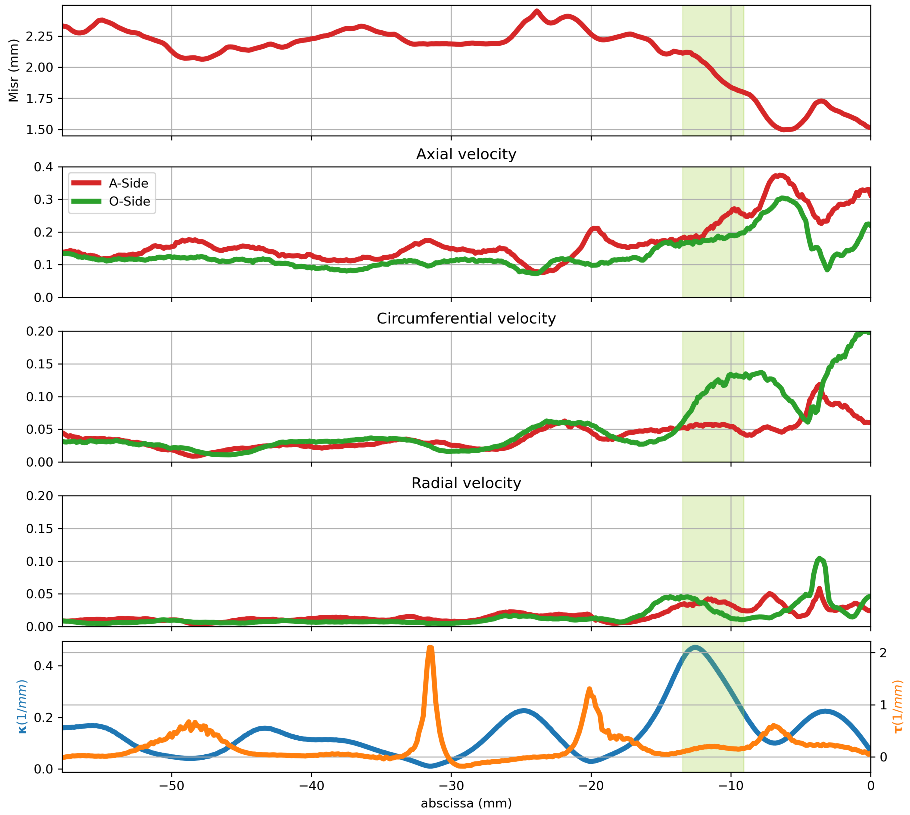

Figure A24: Composite diagrams of case N501.

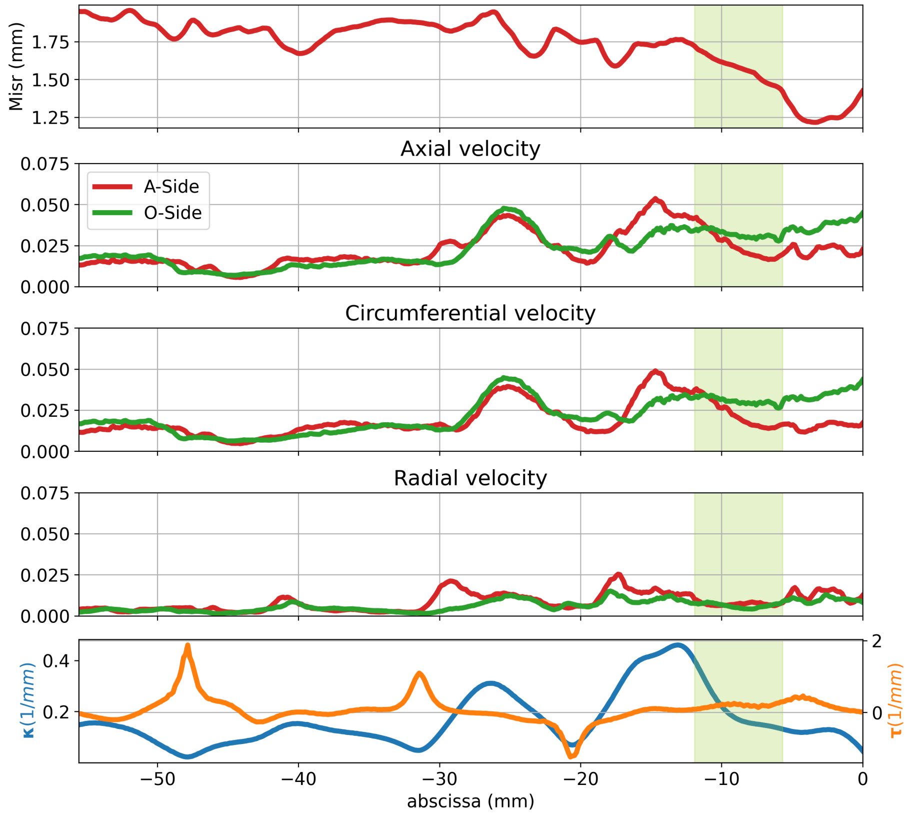

Figure A25: Composite diagrams of case N502.

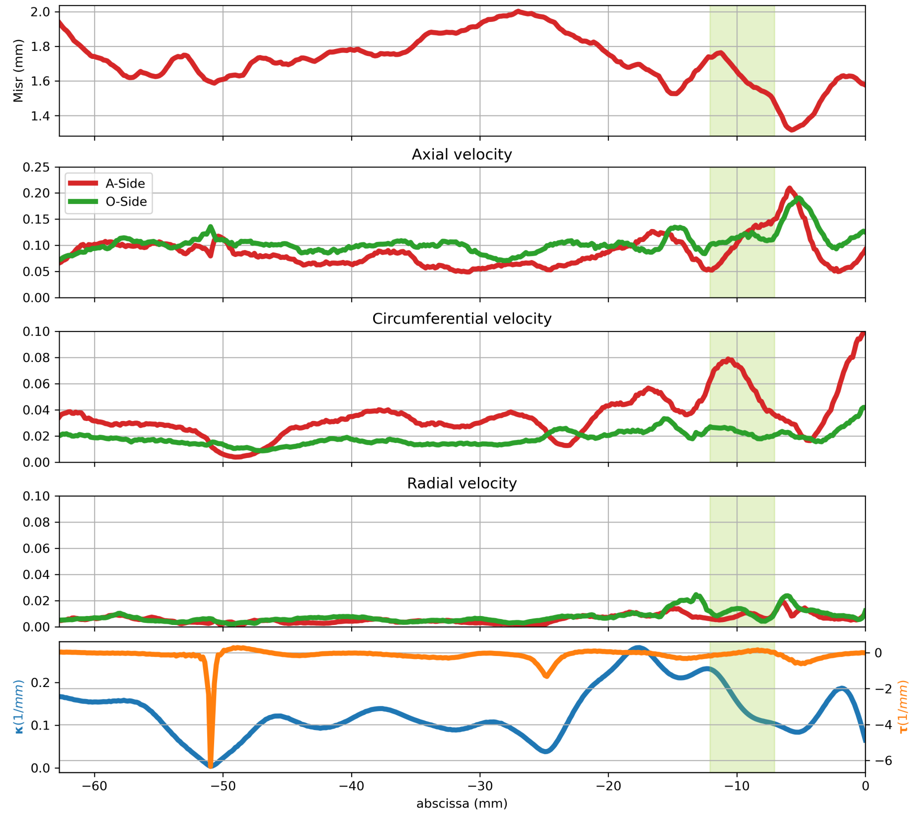

Figure A26: Composite diagrams of case N503.

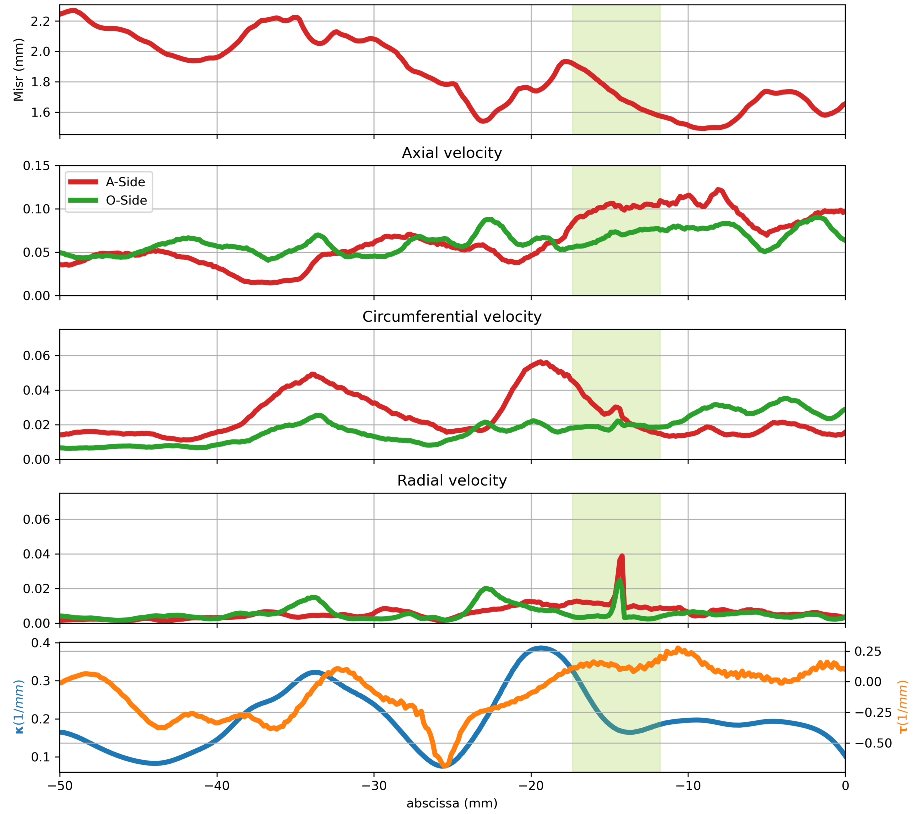

Figure A27: Composite diagrams of case N601.

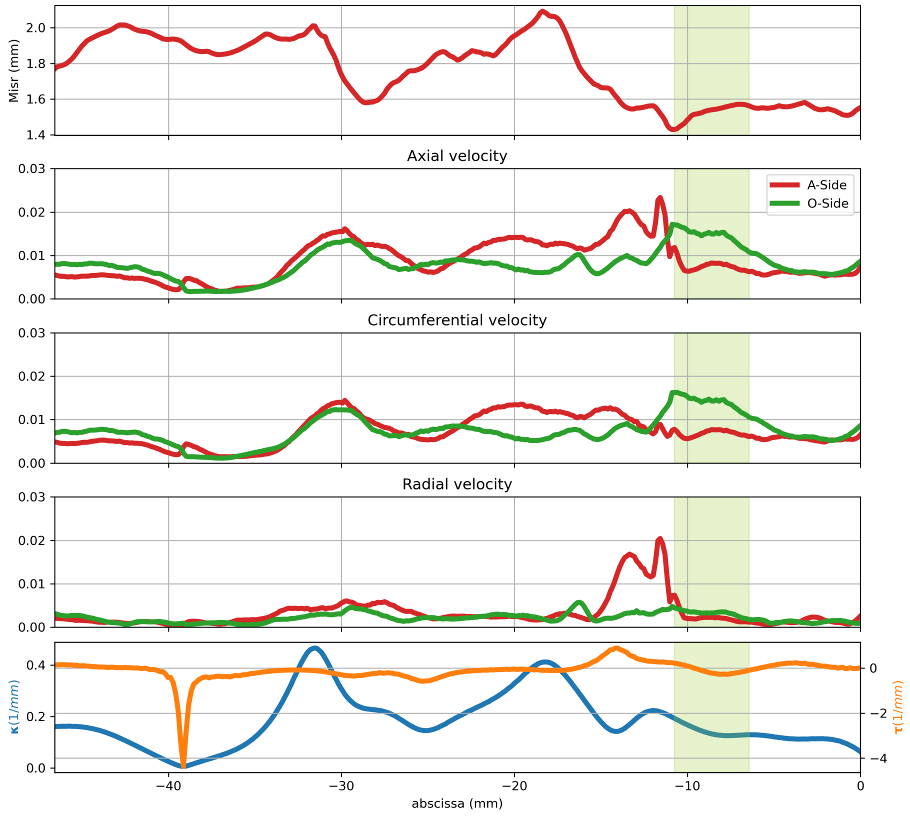

Figure A28: Composite diagrams of case N602.

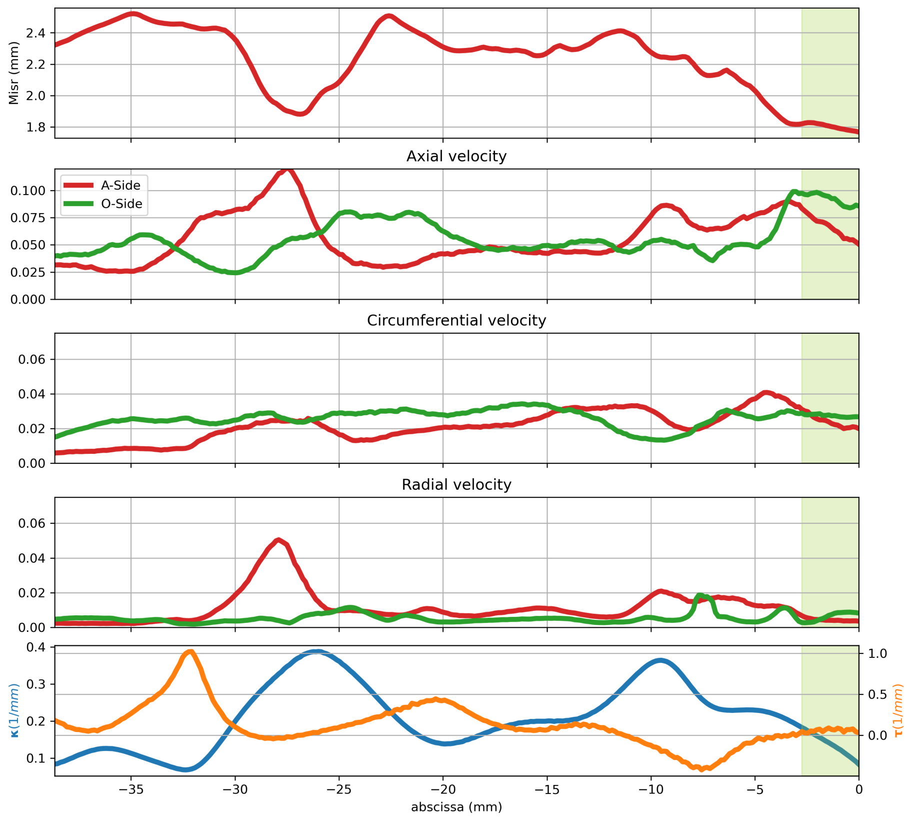

Figure A29: Composite diagrams of case N603.

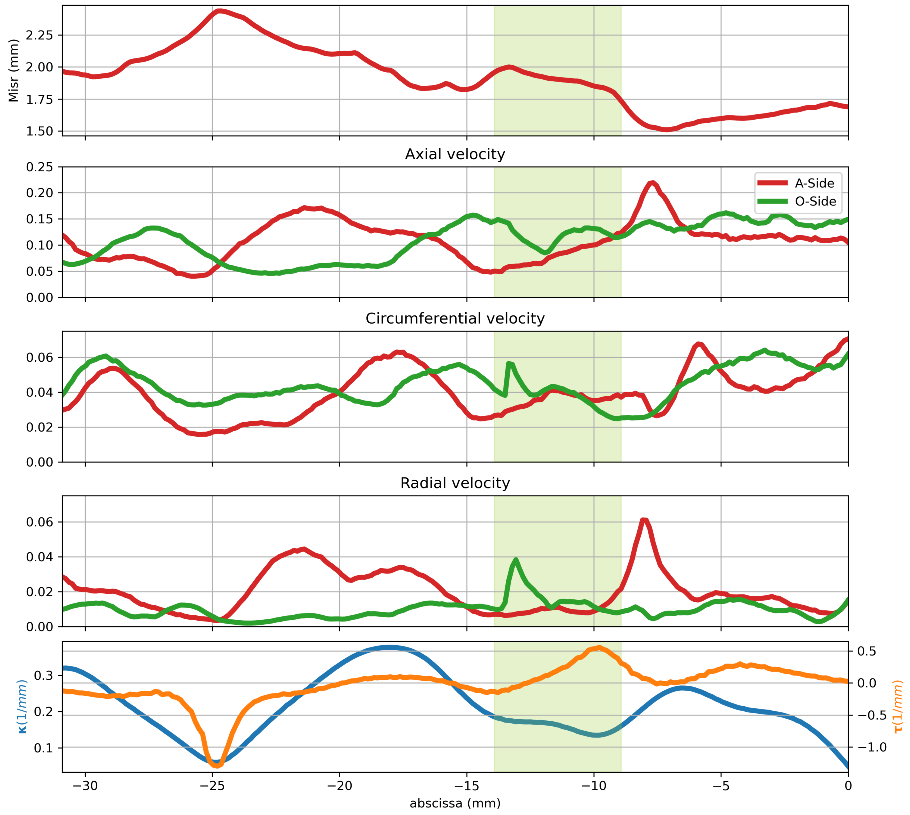

Figure A30: Composite diagrams of case N604.
